# Single photon emission and recombination dynamics in self-assembled GaN/AlN quantum dots

**DOI:** 10.1038/s41377-022-00799-4

**Published:** 2022-04-28

**Authors:** Johann Stachurski, Sebastian Tamariz, Gordon Callsen, Raphaël Butté, Nicolas Grandjean

**Affiliations:** 1 Institute of Physics, École Polytechnique Fédérale de Lausanne, EPFL, CH-1015 Lausanne, Switzerland; 2 Present Address: Université Côte d’Azur, CNRS, CRHEA, F-06560 Valbonne, France; 3 Present Address: Institut für Festkörperphysik, Universität Bremen, 28359 Bremen, Germany

**Keywords:** Quantum dots, Single photons and quantum effects

## Abstract

III-nitride quantum dots (QDs) are a promising system actively studied for their ability to maintain single photon emission up to room temperature. Here, we report on the evolution of the emission properties of self-assembled GaN/AlN QDs for temperatures ranging from 5 to 300 K. We carefully track the photoluminescence of a single QD and measure an optimum single photon purity of *g*^(2)^(0) = 0.05 ± 0.02 at 5 K and 0.17 ± 0.08 at 300 K. We complement this study with temperature dependent time-resolved photoluminescence measurements (TRPL) performed on a QD ensemble to further investigate the exciton recombination dynamics of such polar zero-dimensional nanostructures. By comparing our results to past reports, we emphasize the complexity of recombination processes in this system. Instead of the more conventional mono-exponential decay typical of exciton recombination, TRPL transients display a bi-exponential feature with short- and long-lived components that persist in the low excitation regime. From the temperature insensitivity of the long-lived excitonic component, we first discard the interplay of dark-to-bright state refilling in the exciton recombination process. Besides, this temperature-invariance also highlights the absence of nonradiative exciton recombinations, a likely direct consequence of the strong carrier confinement observed in GaN/AlN QDs up to 300 K. Overall, our results support the viability of these dots as a potential single-photon source for quantum applications at room temperature.

## Abstract (web summary)

Single-photon emission from a single GaN/AlN quantum dot induced by continuous-wave laser excitation, leading to an optimum *g*^(2)^(0) = 0.17 at room temperature.

## Graphical abstract

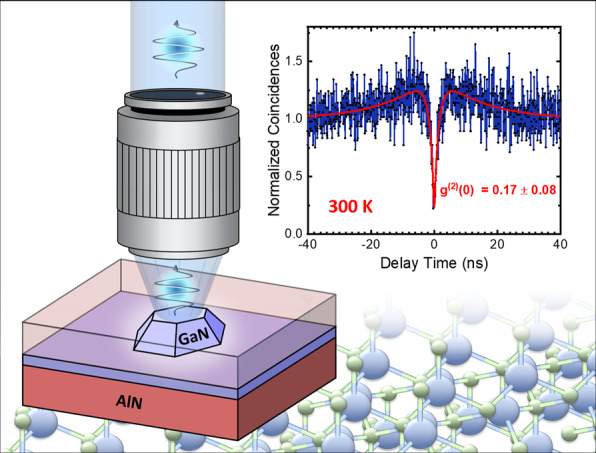

## Introduction

In the framework of the second quantum revolution^1^, single photon emitters (SPEs) have emerged as an important building block for the implementation of fast operating quantum devices. For such use, an ideal SPE should allow for the production of bright photon streams of high purity along with on-chip integration capabilities for industrial purposes. To date, there have been promising candidates in a wide range of solid-state systems^2^, e.g., silicon or nitrogen vacancies in diamond^3–5^, but also other localized defects in 2D^6,7^ and 3D^8,9^ semiconductor materials, as well as semiconductor quantum dots (QDs)^10–12^.

While the development of III-nitride (III-N) QDs for SPE purposes is partially hindered by the dephasing induced by strong phonon^13^ and point defect^14,15^ coupling in wurtzite QDs, recent research has revealed structures in which the impact of point defects is significantly reduced^16^. Weaker dephasing can also be tailored either via a reduction in the QD size^17,18^ or the absence of any built-in electric field in zinc-blende III-N QDs^19,20^. In addition, III-N QDs remain of utmost relevance for room temperature (RT) SPE applications, where their state of the art III-arsenide counterparts are inoperable^21^. III-N QDs may be particularly well suited for quantum operations that do not require photon indistinguishability^2,22^, such as quantum-key distribution^23,24^, quantum imaging^25^ or quantum metrology^26^. Furthermore, the III-N system benefits from an already well-developed infrastructure resulting from its wide use for solid-state lighting applications, which translates into excellent epitaxial growth control and efficient bipolar doping^27^. These examples of appealing properties render the III-N QD platform well adapted for broadband applications with QD emission ranging from the deep ultraviolet down to the infrared^28–31^. In this regard, to date, RT single-photon emission based on III-N quantum heterostructures has already been demonstrated with both GaN/AlGaN^32,33^ and GaN/AlN QDs^34^.

In the present work, we report on several SPE features up to RT of polar self-assembled (SA) GaN/AlN QDs grown on cost-effective Si(111) substrates that are potentially suitable for future on-chip integration. These QDs have been characterized using micro-photoluminescence (µ-PL) measurements under quasi-resonant excitation that were complemented by an analysis of their photon emission statistics. Second-order autocorrelation function (*g*^(2)^(*τ*)) measurements have been carried out on several QDs as a function of excitation power density from 5 to 300 K and analyzed in the framework of a multiexcitonic model. This allowed us to evaluate the impact of thermal broadening on the single photon purity as well as the limits of the adopted framework.

Furthermore, in order to clarify the origin of the long exciton decay times extracted from *g*^(2)^(*τ*) measurements, we performed complementary time-resolved photoluminescence (TRPL) experiments on an ensemble of QDs issued from the same sample over the same temperature range. We specifically investigated the low excitation regime and confronted the observed bi-exponential decays to the current understanding of exciton dynamics occurring in polar SA GaN/AlN QDs.

This article is structured as follows. In Sec. “Main electronic features of polar III-nitride self-assembled quantum dots”, we first recall some specific features of polar SA GaN/AlN QDs with a focus on their main electronic properties described in the framework of the hybrid biexciton model^35^ which is considered to explain the µ-PL results obtained on such single QDs. Then, in Sec. “Framework of the single photon emission measurements” we detail the multi-excitonic model that successfully accounts for the second order autocorrelation measurements performed on single QDs. In this regard, we also provide in Sec. “Single photon emission from self-assembled quantum dots” a full description of the employed *g*^(2)^(*τ*) fitting function as well as an analysis of the experimental *g*^(2)^(*τ*) traces collected over two single QDs from 5 K to RT. In Sec. “Recombination dynamics in a quantum dot ensemble”, we give an overview of the time-dependent photoluminescence features of GaN/AlN QDs as opposed to conventional non-polar SPEs, before providing an in-depth analysis of the temperature- and power-dependent behavior of exciton recombination in Sec. “Exciton recombination processes”. To this end, we propose different scenarios to account for the observed multi-exponential decay and discuss their respective limits. In Sec. “Review of excitonic decay in SA GaN/AlN QDs”, we compare the exciton lifetimes extracted from TRPL transients to former results reported on SA GaN/AlN QDs of both experimental and theoretical nature. We finally conclude in Sec. “Discussion”. Details about the QD growth procedure and the sample preparation for performing single dot spectroscopy are provided in Sec. “Materials and methods”, along with a brief mention of some relevant aspects regarding optical spectroscopy that are further detailed in the supplementary information (SI).

## Results

### Main electronic features of polar III-nitride self-assembled quantum dots

**Fig. 1:**
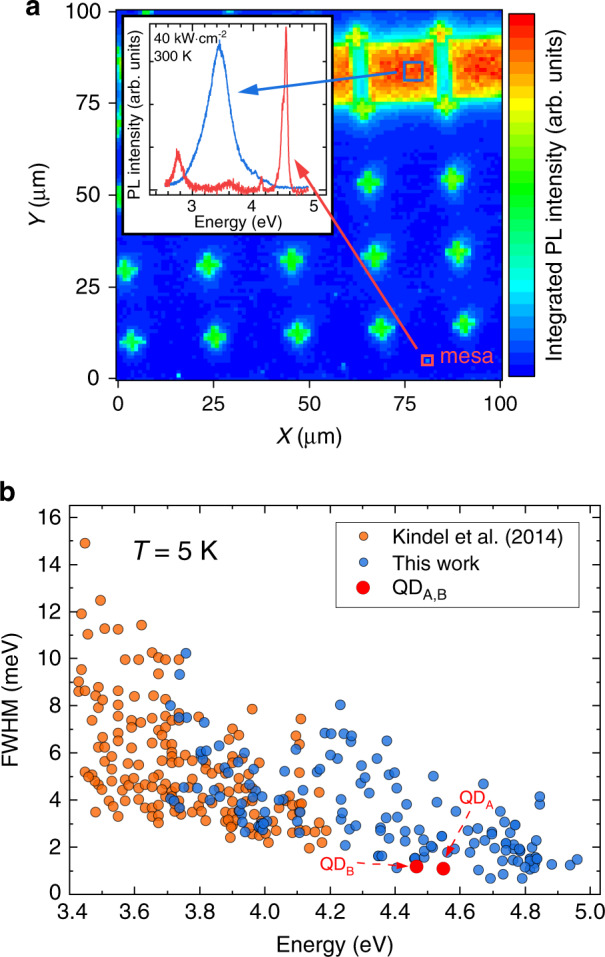
Photoluminescence characteristics of the SA GaN/AlN QD sample. **a** 100 × 100 µm^2^ micro-photoluminescence mapscan of the patterned sample for which the signal is recorded at 300 K and integrated between 2.5 and 4.5 eV. Spatially resolved micro-photoluminescence integrated intensity images are recorded using steps of one µm. The strongest signal is measured from unprocessed areas with a high QD density. Inset: Typical µ-PL spectrum collected from an unprocessed area (blue line) and from a mesa (red line). **b** Full width at half-maximum (FWHM) distribution of GaN/AlN excitonic emission recorded at low temperature (*T* = 5 K) in the cw low excitation regime. Orange dots correspond to QDs grown by metalorganic vapor-phase epitaxy on SiC studied by Kindel et al.^18^. All blue data points were extracted from the same sample. Autocorrelation measurements described in the following are performed on the QDs labeled QD_A_ and QD_B_

All the measurements reported in the present work were carried out on SA QDs grown by molecular beam epitaxy (MBE). The sample surface was patterned into mesa structures to ease the investigation of single QDs. Aspects related to the growth and subsequent processing of the sample are detailed in “Materials and methods”, along with the quasi-resonant excitation scheme. The QD density varies between a few to about one hundred QDs per mesa. The vast majority of the dots emits around 3.5 eV at 300 K, as can be observed by recording µ-PL spectra over unprocessed areas (Fig. 1a). These SA GaN/AlN QDs have a well-known truncated hexagonal pyramid shape inherited from the wurtzite crystal symmetry^36–38^. Owing to the strong built-in electric field that arises along the polar *c*-axis, trapped electrons get pushed toward the top of the pyramid while holes sit at the bottom of the dot^37,38^. The strain induced by the GaN-AlN lattice mismatch results in a piezoelectric field component that leads to an additional lateral confinement of electron and hole wave functions^37,39^. The larger the QD height, the larger the Stark shift, while the quantum confinement is weakened. As a result, the exciton emission energy is redshifted. This quantum-confined Stark effect (QCSE) is experienced without any external electric field. Simultaneously, the electron-hole wave function overlap gets significantly reduced, hence leading to radiative lifetime variations by several orders of magnitude for a QD height increased by a few nanometers only^40,41^.

The strong dipole moment that stems from the electron-hole wave function separation also causes the emission energy of excitonic states to be quite sensitive to the electronic environment. Therefore, GaN/AlN QD PL lines are observed to broaden way above the natural linewidth of excitonic states, which amounts to a few μeV. While this appears detrimental for quantum applications at cryogenic temperature, it can be used to estimate the density of defect states in the vicinity of QDs (see SI Sec. [S4-S5] for further details). Smaller QDs are less impacted by such perturbations, as illustrated by the decrease in the exciton linewidth with increasing emission energy (Fig. 1b). On the other hand, carriers confined in GaN/AlN QDs experience a large trapping potential, which stems from the very thin GaN wetting layer (WL) thickness and the large band offset between AlN and GaN binary compounds. This ensures that high-energy GaN/AlN QDs remain efficient optical emitters up to RT with a large energy separation between confined energy levels.

**Fig. 2:**
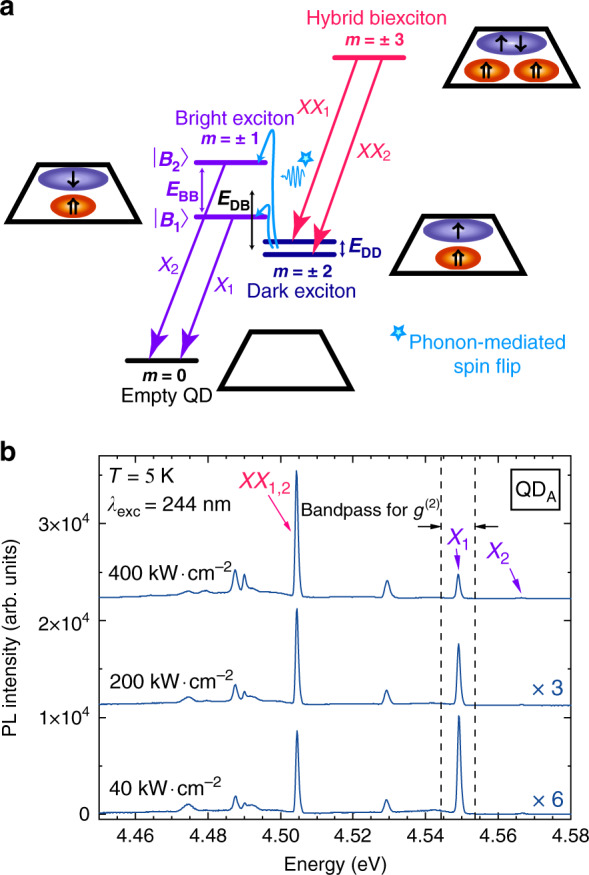
Exciton-biexciton photoluminescence from SA GaN/AlN QDs. **a** Schematic illustration of the hybrid exciton-biexciton cascade. **b** Power-dependent µ-PL spectra of QD_A_ exhibiting X_1,2_ and XX_1,2_ transitions recorded at 5 K

The SA QDs we studied display a large base of a few tens of nanometers^42,43^ and a height of 1 to 3 nm. As a result of this low aspect ratio, the current picture of the electronic energy levels for these dots stands as follows: when two electron-hole pairs lie in the QD ground state, a pronounced in-plane spatial separation of the biexciton (XX) hole wave functions at the QD bottom prevails, which is enhanced by the large effective hole mass^44^. This wave function separation favors the XX configuration with parallel hole spins, which results in a huge binding energy that can reach tens of meV^35^. This XX is referred to as a hybrid-XX and has a total angular momentum *m* = ±3. The exciton ground state splits into two bright states (*m* = ±1, purple energy levels in Fig. 2a) and two dark states (*m* = ±2, dark blue energy levels in Fig. 2a) whose associated degeneracies are lifted likely due to symmetry reduction induced by QD in-plane elongation^45^. Fine structure splittings of a few meVs are usually observed in the polar SA GaN/AlN QD system^35,46^ as a consequence of the strong built-in electric field^47^. The dark (*E*_DD_), bright (*E*_BB_) and dark-to-bright (*E*_DB_) state splittings are illustrated in Fig. 2a. *E*_BB_ is readily accessible in µ-PL spectra as it is defined as the splitting between the two cross-polarized excitonic lines X_1,2_ (purple arrows in Fig. 2a) shown in Fig. 2b for the QD labeled QD_A_. The linear polarization degree of X_1,2_ exceeds 90% for all the investigated QDs (See SI Sect. [S3] for more details regarding single QD polarization measurements). The dark state splitting *E*_DD_ is much smaller and can be deduced from the splitting between the biexciton lines XX_1,2_ (pink arrows). In most experimental situations, however, this splitting cannot be energetically resolved and a single biexciton line is observed^35,46^. The dark-to-bright state transition occurs through a phonon-mediated spin-flip process, whose contribution follows a Bose-Einstein distribution^35,48^. Hence, at low temperature, only the low-energy bright state (B_1_) gets significantly populated. For µ-PL results, this translates into a bright X_1_ line and a dim X_2_ line. The recombination of the high-energy bright state (B_2_), however, is characterized by a stronger oscillator strength^35^, and the X_2_ transition takes over with increasing temperature when the phonon bath involved in the dark-to-bright state transition gets populated (see Fig. S5 in the SI for further details). Additional QD emission lines are commonly observed in µ-PL spectra that are still under investigation. A more exhaustive description of the exciton-biexciton cascade can be found in the work by Hönig et al.^35^.

### Framework of the single photon emission measurements

The complexity of the multi-excitonic recombination scheme led us to methodically adapt the second-order autocorrelation *g*^(2)^(*τ*) function used to describe our results. Indeed, the customary two-level second order correlation function formula *g*^(2)^(*τ*) = 1 − *e*^−(Π+*γ*)·*τ*^ can only be applied under low excitation conditions, for which the pump rate (Π) is smaller than the recombination rate (*γ*)^49^. Under higher excitation conditions, the fast relaxation process of excitons into the QD leads to a bunching phenomenon^50^ that can be explained by the interplay of multi-carrier states: the QD is on average populated with several electron-hole pairs, so that the probability to witness the recombination of a single exciton is enhanced shortly after unloading the QD ground state energy levels. This bunching can be well accounted for by considering a simplified model of multi-excitonic processes^49^. In the latter model, transitions from and toward a level |*n*〉 of *n* excitons trapped in the lowest possible QD energy states correspond to the recombination of a single electron-hole pair describing the QD relaxation toward level |*n* − 1〉 with rate *γ*_*n*_ and the capture of an additional electron-hole pair from level |*n* + 1〉 with the pump rate Π^51^, respectively. Assuming that linear scaling of the recombination rates holds, i.e., *γ*_*n*_ = *n*·*γ*_1_ = *n*·*γ*, the second order exciton correlation function can be expressed by a closed form expression^52^ that reads

1 $$g_{\mathrm{X}}^{{{{\mathrm{(2)}}}}}(\tau ) = ( {{\upmu }} \cdot {{{\mathrm{e}}}}^{ - \gamma {{{\mathrm{|}}}}\tau {{{\mathrm{|}}}}} ) \cdot ( {{{{\mathrm{1-}}}}e^{ - \gamma {{{\mathrm{|}}}}\tau {{{\mathrm{|}}}}}} )$$

where *µ* = Π*/γ* is the QD mean occupation number.

In practice, excitonic states lying above the biexciton one are not observed in µ-PL spectra upon increasing excitation power density for small QDs emitting above 4 eV. This could be explained by a saturation of the pump rate. This is modeled by considering the capture time of an exciton (1*/*Π) as the sum of the time needed to trap an exciton into an excited state, *τ*_t_, and the relaxation time toward the exciton ground state (*τ*_r_)^53^. Variations in the excitation power density *P*_exc_ are only impacting the trapping time, such that 1*/τ*_t_ = *α*·*P*_exc_, where *α* accounts for the pumping efficiency. The mean occupation number can thus be fitted as a function of excitation power density using a relationship given by:

2 $$\mu \left( {P_{{{{\mathrm{exc}}}}}} \right) = \frac{{\mu _{{{{\mathrm{sat}}}}}}}{{1 + P_{{{{\mathrm{sat}}}}}/P_{{{{\mathrm{exc}}}}}}}$$

with *µ*_sat_ = (*τ*_r_·*γ*)^−1^ and *P*_sat_ = (*α*·*τ*_r_)^−1^ being two fitting parameters, which leads to the expected linear dependence for low excitation conditions. Let us note here that Auger-assisted relaxation processes^54,55^ that could decrease *τ*_r_ are not considered in this framework. Indeed, the latter phenomenon can be expected to be relatively weak considering our quasi-resonant excitation scheme for which interactions with excited carriers in the WL are unlikely. As a result nonradiative Auger recombination processes have not been reported in GaN/AlN QDs so far. However, we cannot fully exclude its contribution to account for the saturation of *µ* and the quenching of multi-excitonic states.

In addition to the impact of multi-excitonic states on photon statistics described above, spectral jittering of the exciton line occurring on the nanosecond to picosecond timescale could induce some additional photon bunching in the *g*^(2)^(*τ*) traces^56,57^. The amplitude of this bunching depends on the overlap between the emission line and the detection window and cancels out when the former is fully encompassed in the latter^58^. Thanks to the large energy spacing between the QD emission lines and the low background noise level, we could successfully avoid the impact of spectral wandering at low temperature by making use of an 8 meV detection bandpass. The exciton line, whose linewidth amounts to 1 meV, is thus fully encompassed in this bandpass, as illustrated in Fig. 2b. At higher temperatures, the line broadening is mainly driven by phonon coupling occurring on a picosecond timescale, i.e., below the setup detection limit^59–61^. We can therefore expect spectral wandering of the exciton line to have no significant impact on the determination of the second order correlation function, regardless of the temperature.

Finally, we point out that the experimental fitting function also accounts for the instrument response function (IRF) such that

3 $$g_{{{{\mathrm{exp}}}}}^{{{{\mathrm{(2)}}}}}{{{\mathrm{(}}}}\tau {{{\mathrm{)}}}} = g_{\mathrm{X}}^{{{{\mathrm{(2)}}}}}{{{\mathrm{(}}}}\tau {{{\mathrm{)}}}} \otimes {{{\mathrm{IRF(}}}}\tau {{{\mathrm{)}}}}$$

The instrument response becomes detrimental only when the characteristic antibunching time approaches the time resolution of 220 ps.

### Single photon emission from self-assembled quantum dots

**Fig. 3:**
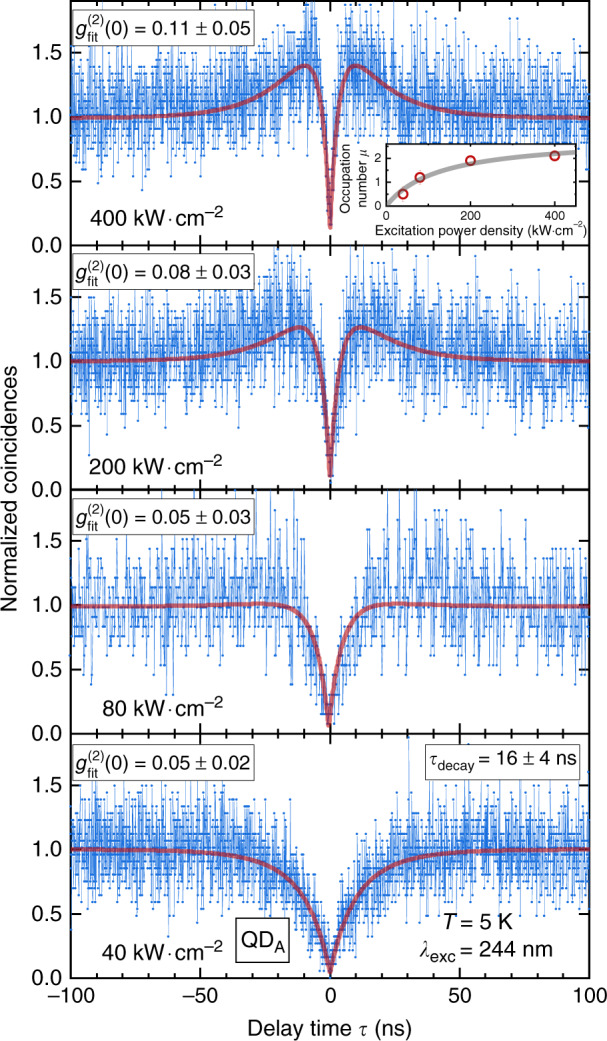
*g*^(2)^(*τ*) traces of QD_A_ recorded at 5 K as a function of excitation power density (connected blue diamonds) and convoluted fits (red lines). The channel resolution is fixed to 100 ps/channel at 40, 200 and 400 kW cm^−2^. Given the smaller number of total coincidences measured at 80 kW cm^−2^, the corresponding data are shown with a 200 ps/channel resolution. The exciton decay time being in theory power-independent, its value is extracted by fitting all traces with a shared *τ*_decay_ value. The inset shows the evolution of the fitting parameter *µ* upon increasing power. The data (red circles) are fitted using Eq. 2 (grey line,) which yields *µ*_sat_ = 2.9. All uncertainties are obtained in the simplified framework described in the text and indicate the robustness of the fit. The full collected data span a range of ±130 ns around 0

*g*^(2)^(*τ*) traces recorded at various excitation power densities at *T* = 5 K are displayed in Fig. 3. They all show a clear antibunching at zero delay time and a signature of the above-mentioned bunching phenomenon resulting from multi-exciton state occupancy under high excitation conditions. The purity determined with the fitting function (*g*^(2)^_fit_) is also given. The *g*^(2)^(*τ*) data are fitted using Eq. 3 with a fixed exciton decay time *τ*_decay_ = *γ*^−1^ = 16 ± 4 ns deduced by fitting all traces with a shared recombination rate *γ*. This value exceeds by about one to two orders of magnitude the decay times previously reported for GaN QDs emitting above the bulk GaN bandgap^62–65^. First, this suggests a very low nonradiative recombination rate. This is consistent with the fact that the QD of interest remains bright despite such a long-lived recombination time for the excitons. This comparatively long exciton decay time may originate from a transfer toward the dark states depicted in Fig. 2a, that could act as a long-lived reservoir in the absence of any efficient thermally enhanced phonon mediated spin flip between dark and bright states^66,67^. The single photon purity is observed to decrease with rising excitation power density, as a consequence of the temporal resolution. At 40 kWcm^−2^, the measured purity of 0.05 ± 0.02 compares well to the lowest values measured to date in III-N systems, namely GaN QDs formed at step edges in low aluminum content GaN/AlGaN quantum wells^16^. The narrowing of the antibunching dip can be explained based on an increase in the mean occupation number *µ*, along with the pump rate. The sublinear pump power dependence of *µ* is shown in the inset of Fig. 3 and highlights the saturation of the QD filling. Let us note, however, that the fitting of the antibunching dip (red lines shown in Fig. 3) with a global variable *γ* differs from the results obtained when fitting each *g*^(2)^(*τ*) trace individually. The decay time is especially affected by small variations in the mean occupation number, as a result of the exponential dependence in *µ* of the bunching (Eq. 1). This translates into a large uncertainty obtained for *τ*_decay_ and a slight discrepancy between fitted and experimental traces. Variations in the measured exciton decay time could originate from power-dependent fluctuations of the built-in electric field. Such changes, ascribed to variations in the charging dynamics of defects surrounding the QDs^52^, are, however, expected to be negligible for QDA due to its high emission energy (see details in the SI). Alternatively, spin flip processes between dark and bright states could also account for changes in the exciton lifetime. A more realistic model would require to consider transitions rates between nondegenerate exciton and biexciton states that would add to the current number of unknown variables.

**Fig. 4:**
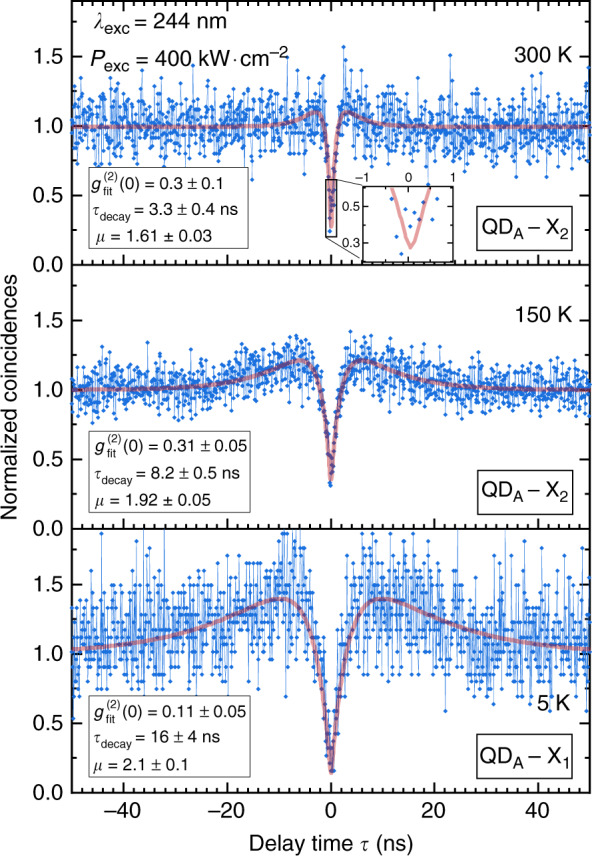
*g*^(2)^(*τ*) traces of QD_A_ recorded at an excitation power density of 400 kW cm^−2^ as a function of temperature, with a channel resolution of 100 ps/channel (connected blue diamonds) and convoluted fits (red lines). All uncertainties are obtained in the simplified framework described in the text and only indicate the fitting robustness. The full collected data span a range of ±130 ns around 0. The inset in the top panel is a zoomed-in view of the antibunching dip

In the following, we focus on the temperature-dependent behavior of SPE features exhibited by these SA GaN/AlN QDs. For such dots, it has been shown that the antibunching amplitude can display a progressive decrease above cryogenic temperature and cross the SPE limit (*g*^(2)^(0) ≥ 0.5) under the increased contribution of spurious background signals^68^. However, the *g*^(2)^(*τ*) traces shown in Fig. 4, measured successively on the X_1_ (5 K) and X_2_ lines (150 K and 300 K), underline the remarkable conservation of the single-photon purity from 5 K up to RT for QD_A_. This can be explained by the large spectral separation between the exciton and the biexciton emission lines (see, e.g., Fig. 2b), such that the signal collected within the detector bandpass remains dominated by the exciton emission, despite the increasing weight of thermal broadening. The observed narrowing of the antibunching dip with increasing temperature is expected to result from both an increase in the exciton decay rate, as a consequence of both faster nonradiative recombination processes, and more presumably an increase in the pump rate as the phonon-assisted relaxation time *τ*_r_ is reduced. In addition, in the specific case of these dots, the enhancement of the decay rate between 5 and 150 K is also explained by the larger oscillator strength of the X_2_ transition that takes over the X_1_ line with increasing temperature due to thermal population^35^.

**Fig. 5:**
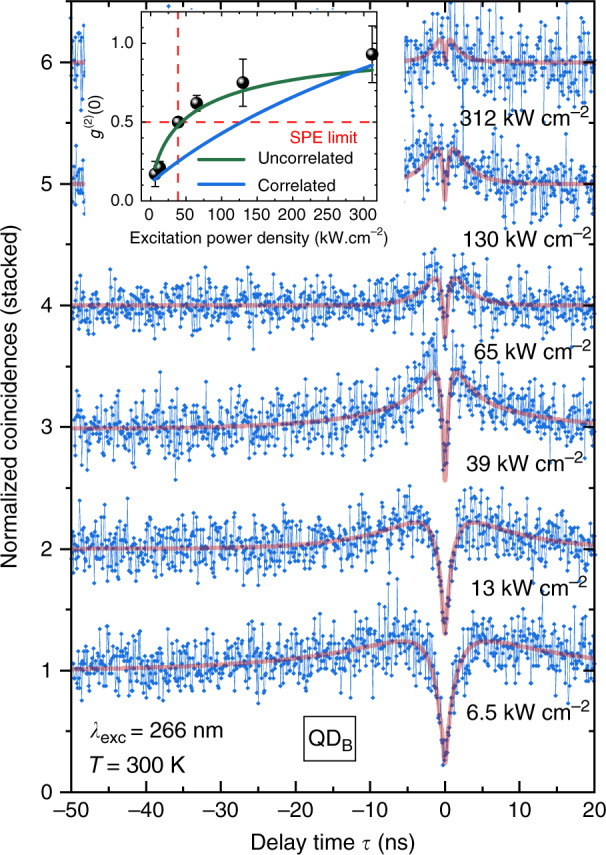
*g*^(2)^(*τ*) traces of QD_B_ recorded at 300 K as a function of excitation power density with a channel resolution of 100 ps/channel (blue connected diamonds) and convoluted fits (red lines). Inset: evolution of the single-photon purity with rising excitation power density where the solid lines are the results issued from models assuming either an uncorrelated background (green line) or a correlated one (blue line). Adapted from Tamariz et al.^34^

The high single photon purity observed for QD_A_ at RT is not an isolated case and has been measured for different QDs we investigated^34^. To strengthen this point, Fig. 5 displays the evolution of another SPE, labeled QD_B_, upon variation of the excitation power density, with a RT purity of *g*^(2)^(0) = 0.17 ± 0.08 at the lowest reported excitation power density of 6.5 kW cm^−2^. The antibunching amplitude is again observed to decrease upon increasing excitation power density (see the inset of Fig. 5), which is in line with the superlinear increase in the biexciton intensity and its resulting stronger contribution to the recorded *g*^(2)^(*τ*) signal. Let us point out that the contrasting responses to excitation at equivalent power densities displayed by QD_A_ and QD_B_ could originate from strong changes in the absorption coefficient of such 0D nanostructures when excited at two different quasi-resonant excitation energies (4.66 and 5.08 eV).

This loss in single photon purity is included in Eq. 3 by introducing an offset parameter *g*^(2)^(0) that accounts for the contribution of parasitic background emission. As previously mentioned, at RT, the strongest contribution to this background emission stems from the broadened XX emission line, which is overlapping with the detection window. The inset of Fig. 5 shows the expected evolution of the single-photon purity as a function of excitation power density, where the blue line is a theoretical estimate of *g*^(2)^(0) when summing up all second-order auto- and cross-correlation terms involving X and XX^34^. The observed loss in single-photon purity is eventually more properly described by considering the XX luminescence as an uncorrelated background (green line in the inset of Fig. 5) along the lines given in ref. ^69^:

4 $$g^{{{{\mathrm{(2)}}}}}(0) = {{{\mathrm{1}}}}-\rho ;\rho = \frac{{I_{\mathrm{X}}}}{{I_{\mathrm{X}} + I_{{{{\mathrm{XX}}}}}}}$$

**Fig. 6:**
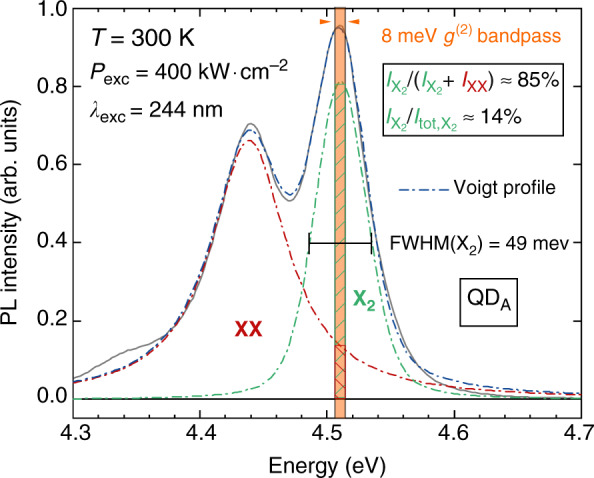
Room temperature µ-PL spectrum recorded on QD_A_ approximated by a two-peak function. The biexciton is fitted with a Lorentzian (red dash-dotted line). X_2_ is not fully Lorentzian and is best approximated by a Voigt function (green dash-dotted line), hinting at the underlying presence of X_1_ whose impact remains however negligible. The resulting two-peak fit is highlighted with a blue dash-dotted line. The detection window of the HBT setup is indicated along with the contribution of X_2_ (*I*_X2_) and XX (*I*_XX_) to the measured signal (hatched areas). *I*_tot,X2_ stands for the integrated intensity of the X_2_ line. The FWHM of X_2_ is highlighted

*I*_X_ and *I*_XX_ correspond to the intensity of the X and the XX lines within the bandpass of the Hanbury-Brown and Twiss (HBT) interferometer setup (see “Materials and methods”), that are directly deduced from the related µ-PL spectra as depicted in Fig. 6. At 400 kW cm^−2^, the signal-to-noise ratio *ρ* ≃ 0.85 measured on QD_A_ would lead to a single photon purity of 0.27, a value in very good agreement with experimental observations (Fig. 4). In this respect, a large exciton-biexciton splitting appears crucial to ensure single-photon emission at RT. The low *g*^(2)^(0) value we report is explained by the X_2_-XX splitting we measure that ranges from 62 meV (5 K) to 72 meV (300 K) for QD_A_. Let us note that this exciton-biexciton splitting is larger than the biexciton binding energies previously reported for GaN/AlN QDs^11^, which most likely stems from a particularly small QD aspect ratio (∼0.1) known to result in a large positive biexciton binding energy (*E*_X_ − *E*_XX_ > 0)^35^. Qualitatively, a large aspect ratio (large QD diameter) leads to a reduced pairwise Coulomb repulsion, while the electron-hole exchange interaction is enhanced for small QDs^70^. On this basis, the observed energy variations in the biexciton binding energy with temperature could tentatively be ascribed to a complex interplay between the respective weight of direct Coulomb interaction and exchange, as well as correlation interactions experienced by trapped carriers upon lattice expansion. While the *g*^(2)^(*τ*) spectral bandpass of our HBT setup is limited to a maximum value of 8 meV, the collection efficiency of the exciton photoluminescence could be improved by making use of a larger detection window while causing little deterioration to the single-photon purity. As an illustration, *ρ* would be reduced from 85 to 82% for QD_A_ at an excitation power density of 400 kW cm^−2^ (Fig. 6) for an increase in the *g*^(2)^(*τ*) bandpass from ∆*E* = 8 meV to ∆*E* = 49 meV, the latter value corresponding to the exciton linewidth reported at RT. Hence, the corresponding 5% loss in single photon purity would result in a 5 times brighter signal. More details are provided in the SI Sec. [S6].

The large spectral separation between the exciton and the biexciton lines and the high exciton binding energy of GaN/AlN QDs make them particularly suited for RT single-photon emission, as highlighted by the single photon purity *g*^(2)^(0) = 0.17 ± 0.08. In this regard, the role of the very thin WL, with a high electronic state energy of about 5.3 eV (i.e., 234 nm), is also crucial. Indeed, the 244 nm and 266 nm continuous-wave lasers used to pump the structure generate electron-hole pairs directly into the QDs, hence avoiding any spurious signal from the WL.

### Recombination dynamics in a quantum dot ensemble

**Fig. 7:**
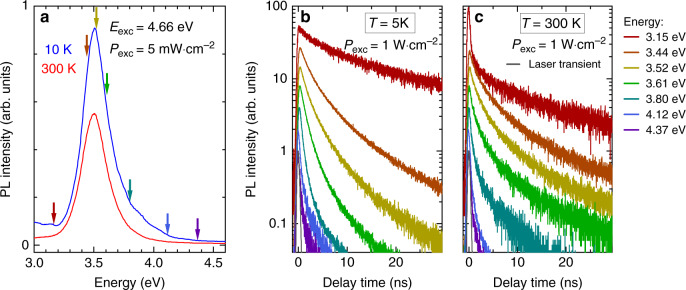
Time-resolved photoluminescence from a QD ensemble. **a** Temperature dependence of the QD ensemble photoluminescence. The arrows mark the energies at which the TRPL transients shown in **b** and **c** are measured. The excitation power density is chosen low enough to ensure that most of the signal stems from excitonic recombination. Time-resolved photoluminescence transients measured at 5 K **b**. and 300 K **c**. are displayed. The laser transient is also given (grey line)

While robust RT single-photon emission has been demonstrated for these SA GaN/AlN QDs, the long exciton decay time of 16 ± 4 ns extracted from second-order correlation function measurements recorded at 5 K on QD_A_, whereas it is emitting near 4.5 eV, is not in line with previous experimental results^62–65^. Therefore, in order to get a more general and more conclusive picture on the exciton dynamics in this system, we performed TRPL measurements on an ensemble of SA QDs by probing an unprocessed area on the sample. PL transients were collected for temperatures ranging from 5 to 300 K and excitation power densities covering the 2 to 1.2 × 10^3^ mW cm^−2^ range, leading to a maximum energy density per pulse of 0.15 mJ cm^−2^. While in the low-density regime the PL spectra of the QD ensemble peak at ∼3.5 eV whatever the temperature (see Fig. 7a), the PL intensity remains large enough to record PL transients of QDs emitting from 3.2 to 4.5 eV.

PL transients measured at 5 and 300 K are shown in Fig. 7b, c for various QD emission energies corresponding to a spectral window that ranges from 4 meV for low energy emitting QDs to 8 meV for high energy ones. At high excitation power density, all transients display a multi-exponential decay profile, which can be intuitively attributed to multi-carrier filling of the QDs. A contribution from the WL can, once again, be discarded as all the dots are pumped quasi-resonantly. Unlike previous observations^65^, the decay rate at short delays is strongly impacted by the QD size. In fact, the initial decay rate is enhanced for high energy emitters, hinting at an increase in the multi-carrier recombination rate due to the larger electron-hole wave function overlap. As a comparison, in colloidal QDs multi-carrier recombination is usually dominated by nonradiative Auger recombination processes occurring on a ps timescale^71^. On the other hand, Auger processes are commonly considered as negligible in self-assembled InAs/GaAs QDs and were only recently observed to occur on a timescale on the order of a few microseconds^72^. As such, multicarrier recombination rates can be described assuming a pure radiative recombination process^73^. We initially attempted to account for TRPL data in this framework, assuming the same linear scaling of the recombination rates (*γ*_*n*_ = *n*·*γ*_1_ = *n*·*γ*) than that considered to depict SPE results. However, when applying this approach to a QD ensemble the increasingly fast decay observed at high excitation power density could not be satisfactorily reproduced. This discrepancy could be attributed either to Auger processes speeding up the recombination of multi-carrier states or even faster multi-excitonic radiative recombination processes. One can also note that at high excitation and high emission energy the fast decays occur on a timescale on the order of a nanosecond or even below which is close to the laser pulse duration (Fig. 7b, c), hence preventing us from reaching any firm conclusion at this stage.

In addition, we recall that polar III-N QDs differ in a specific manner from their InAs/GaAs counterparts. Indeed, QDs emitting below the bulk GaN bandgap experience a large QCSE leading to a power-dependent Stark shift^64^. This shift can in turn drive different emitters in and out of the detection window. This behavior stems from a progressive descreening of the built-in field when the number of trapped carriers decreases upon increasing time and could in turn additionally explain the failure of the above-mentioned modeling. The impact of the excitation power density on PL spectra is clearly discernible through a spectral shift of the QD ensemble peak energy by up to 50 meV between 2 and 1.2 × 10^3^ mW cm^−2^ (see Fig. S9 in the SI); a shift that vanishes for high energy QDs, which are less affected by the QCSE. Alternatively, QDs emitting above 4 eV exhibit large positive biexciton binding energies (*E*_XX_ < *E*_X_) that can reach a few tens of meV^35^. Hence, the intermixing of different excitonic complexes could potentially occur when collecting TRPL transients at high energy that might affect in some ways the readability of the results.

### Exciton recombination processes

**Fig. 8:**
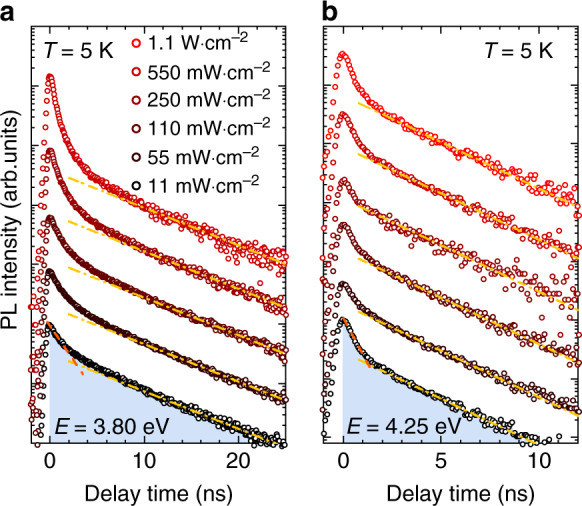
Photoluminescence decays of GaN QDs recorded at 5 K. The transients are given for QDs emitting at **a** 3.80 eV and **b** 4.25 eV, for increasing excitation power densities. The tail of each transient is approximated by a single exponential with a *τ*_decay_ value of 6.5 and 2.8 ns, respectively (yellow dash-dotted lines). At low excitation power density, the decay is bi-exponential. The fast decay component is also highlighted in the graph (orange dashed line). Noticeably, the discrepancy between the bi-exponential fit and the experimental data increases with excitation power density. The channel resolution was reduced down to 80 ps/channel to improve the qualitative appraisal of decay time conservation. As an illustration, the filled areas correspond to the integrated intensity *I*_tot_ collected at 11 mW cm^−2^

Given the above-mentioned complexity of multi-carrier processes, we can first focus on the description of the physics related to the tail of the TRPL transients instead. A long-lived mono-exponential decay is observed at any selected energy, which we associate to single exciton recombinations across the QD ensemble in the following. Figure 8 shows the power-dependent evolution of TRPL transients for QDs emitting at 3.80 and 4.25 eV recorded at a temperature of 5 K. At low excitation power density, the long-lived mono-exponential decay is maintained over a dynamic range exceeding two orders of magnitude. The characteristic decay time (*τ*_decay_) is equal to 6.9 ± 0.3 ns and 2.8 ± 0.2 ns, respectively, (cf. yellow dash-dotted lines in Fig. 8). This long-lived decay is preserved at higher excitation power density and the tail of each transient can be consistently approximated by the same lifetime, as expected for the radiative recombination of excitons emitting at a given energy.

**Fig. 9:**
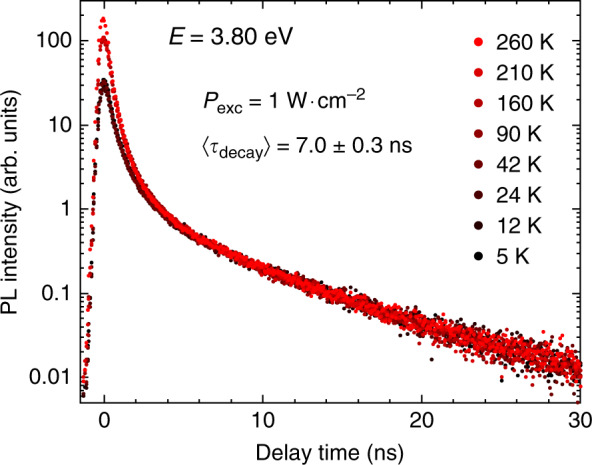
Photoluminescence decays of GaN QDs emitting at 3.80 eV recorded from 5 to 260 K at an excitation power density of 1 W cm^−2^. All curves are stacked in order to superimpose the tail of the TRPL transients. The channel resolution was reduced down to 80 ps/channel to improve the qualitative appraisal of decay time conservation

To further support this picture associated to single exciton decay, low temperature TRPL measurements have been completed by a temperature-dependent series for QDs emitting at the same energy of 3.80 eV (Fig. 9). Interestingly, we do not observe any noticeable change in the decay time extracted from the tail of TRPL transients when heating the sample up to 260 K. Such a behavior is expected for zero-dimensional nanostructures for which the radiative lifetime is independent of temperature^74^. We note that the same observation has been reported on SA GaN/AlN QDs grown on *c*-plane sapphire substrates^75^. This is also consistent with a system free from any nonradiative recombinations.

**Fig. 10:**
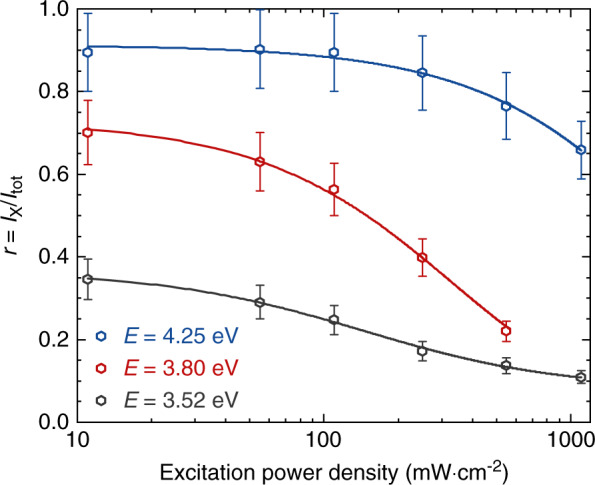
Low temperature (*T* = 5 K) evolution of the *I*_X_*/I*_tot_ ratio as a function of excitation power density for various QD emission energies. Solid lines serve as a guide to the eye

With solid evidence that the tail of the TRPL transients is associated to single exciton decay, we can now compare the weight of this long-lived mono-exponential PL component to that of the whole decay profile at different energies and for varying excitation power densities. The exciton intensity *I*_X_ is estimated by integrating the mono-exponential fit for times following the disappearance of the short lifetime component of each transient. The results are normalized to the integrated intensity *I*_tot_ measured over the whole raw data (*τ* > 0) for each decay profile. The power-dependent trend of *r* = *I*_X_*/I*_tot_ for PL decay traces recorded at *T* = 5 K is given in Fig. 10 for QDs emitting at 3.52, 3.80 and 4.25 eV. The weight of single exciton PL is logically observed to decrease with increasing excitation power density as multi-excitonic recombination gets more likely. This manifests itself as a persisting bi-exponential decay, which does not vanish at low excitation power density (see Fig. 8). However, the asymptotic saturation of *r* toward *r*_0_ upon decreasing excitation power density, which is most noticeable for high energy emitting QDs, is consistent with a picture where the observed PL decays at low power densities do not originate from multi-carrier recombination.

In this regard, transients collected at the lowest *P*_exc_ values can be described using the two-component decomposition:

5 $$I({{{\mathrm{t}}}}) = A_{\mathrm{S}} \cdot e^{ - t/\tau _{\mathrm{S}}} + A_{\mathrm{L}} \cdot e^{ - t/\tau _{\mathrm{L}}}$$

with a short (*τ*_S_) and a long (*τ*_L_) decay term. *A*_S,L_ prefactors are fitting parameters, which can be linked to the ratios *r*_0_ shown in Fig. 10 as follows:

6 $$r_0 = \frac{{\tau _{\mathrm{L}} \cdot A_{\mathrm{L}}}}{{\tau _{\mathrm{S}} \cdot A_{\mathrm{S}} + \tau _{\mathrm{L}} \cdot A_{\mathrm{L}}}} = \frac{{\tau _{\mathrm{L}}}}{{\tau _{\mathrm{S}} \cdot A_{\mathrm{S}}/A_{\mathrm{L}} + \tau _{\mathrm{L}}}}$$

At this stage, one should provide a physical explanation able to connect the short-lived component of PL decay traces to the long-lived one. On the basis of the schematic electronic picture shown in Fig. 2a, two approaches could potentially account for the observed bi-exponential transient. As a first possibility, the biexponential decay could be attributed to interactions taking place between dark and bright states. In this framework, excitons trapped in a bright state can either recombine radiatively, with rate *τ*_S_^−1^, or undergo a spin-flip process toward a dark state. The long-lived decay would thus relate to the reloading from the dark state with characteristic time *τ*_L_^67^. Yet dark-to-bright state transitions are phonon-mediated spin-flip processes. As such, they get enhanced when the number of phonons with high enough energy is increased, i.e., when the temperature increases. In this picture, we expect the reloading time *τ*_L_ to decrease with temperature, down to a point where the exciton decay becomes mono-exponential, when *τ*_L_ ≪ *τ*_S_. This however does not agree with experimental observations, as we do not observe any change whatsoever in *τ*_L_ when heating the sample (see Fig. 9).

Alternatively, we could attribute the bi-exponential decay to exciton radiative recombination from the low energy (*γ*_B1_) and high energy (*γ*_B2_) bright states, respectively. At low temperature, most QD photoluminescence is supposed to originate from B_1_, as reloading from dark states toward B_2_ is thermally suppressed. However, radiative recombination from B_2_ cannot be completely discarded and the fast decay could be interpreted as $\tau_{\text{S}}^{-1} \simeq \gamma_{{\text{B}}_{2}} + \gamma_{{2}\downarrow}$ where *γ*_2↓_ would correspond to the relaxation rate from B_2_ to the dark states.

In this framework, we can foresee an enhancement in the B_2_ luminescence either through a reduction in the bright state splitting *E*_BB_ or through a thermal enhancement in B_2_ reloading. Experimentally, this can be linked to a decrease in *r*_0_, i.e., an increase in the *A*_S_*/A*_L_ ratio. As shown in Fig. 10, *r*_0_ drops from 0.9 at 4.25 eV to 0.37 at 3.52 eV. Such an observation is consistent with the reported reduction in the bright-state splitting *E*_BB_ with decreasing QD emission energy^46^. In the same vein, we observe a drop from *r*_0_ = 0.7 at 5 K to *r*_0_ = 0.5 at RT for QDs emitting at 3.80 eV (see Fig. S10 in the SI), which is consistent with a thermal activation of the short-lived decay signal. However, due to the reduced intensity of the high energy tail of the QD ensemble PL, deriving the temperature-dependence of *r* at various excitation power densities for QDs emitting above 4 eV proved a too challenging task. Nonetheless, we could still observe a drastic drop in the weight of the long-lived emission component of the transients for QDs emitting at 4.25 eV with increasing temperature, as *r* decreased from 0.82 at 5 K down to 0.25 at RT at an excitation power density of 0.35 W cm^−2^. Further details are provided in the SI Sec. [S8].

Although the bi-exponential decay seems to be fairly well accounted for by exciton recombination from the two bright states, several considerations still challenge this hypothesis. First, the bright state splitting is expected to become negligible below 4 eV^46^, with a value dropping below 1 meV. Hence, the refilling of B_2_ should be fully activated for QDs detected at such energies. Conversely, the strong reduction of *r*_0_ we measured from 3.8 to 3.5 eV suggests a weak contribution of B_2_ recombination at 3.8 eV. Secondly, the lift of degeneracy between B_1_ and B_2_ must be accompanied by a convergence of their respective radiative recombination times. In practice, however, *τ*_L_ was consistently measured to be 4 to 5 times larger than *τ*_S_, regardless of the emission energy. From this last observation, it clearly appears that the bi-exponential decay can no longer be solely explained by the recombination of the exciton bright states for low energy QDs. With this in mind, the B_2_ phonon-mediated refilling mechanism described beforehand could also be associated to alternative QD states involving B-hole states. Indeed, so far, we only considered excitonic states for which the hole is located in the A-valence band. A more complete analysis could take into account states originating from both the A- and B-valence bands. The splitting between A- and B-exciton states has been theoretically estimated to amount to more than 10 meV in GaN/AlN QDs, with little dependence on the QD size^76^. Hence, this splitting is large enough to strongly inhibit B-exciton emission at 5 K while allowing its thermal activation at RT. In this perspective, the fast decay time *τ*_S_ can be interpreted as the result of the intermixing of the B-exciton radiative recombination time and its relaxation time toward the A-exciton, even though the latter interpretation still lacks any direct experimental evidence.

At this stage, the analysis we propose here remains only partially satisfactory as it does not allow us to conclude unequivocally on the origin of the short-lived decay. Besides, reconciling the long decay time determined by autocorrelation measurements at 5 K (*τ*_decay_ = 16 ± 4 ns) and the long-lived decays (*τ*_L_(4.5 eV) ≃ 3 ns) extracted from TRPL data remains a daunting challenge, as detailed hereafter.

**Fig. 11:**
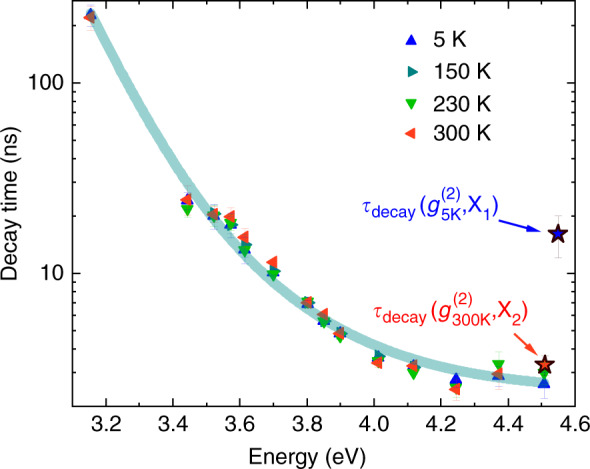
Evolution of the long-lived component decay time *τ*_L_ as a function of energy for temperatures ranging between 5 and 300 K. Decay times extracted from *g*^(2)^(*τ*) traces measured on QD_A_ at 5 K and at RT are also reported for comparison (blue and red stars). The solid line serves as a guide to the eye

As shown beforehand (cf. Fig. 9), the recombination kinetics is essentially unaffected when increasing the temperature, which leads to nearly constant exciton lifetimes up to 300 K, regardless of the emission energy (Fig. 11). However, the same statement does not apply to the decay time extracted from *g*^(2)^(*τ*) traces obtained from QD_A_, which drops from 16 ns at 5 K to 3.3 ns at RT (Fig. 4). While this variation could be ascribed to the difference in oscillator strength between B_1_ and B_2_, the drop in *τ*_decay_ observed between 150 and 300 K –whereas excitonic recombination is supposed to originate from the B_2_ state at these two temperatures– is in clear contradiction with TRPL trends. Part of the explanation could lie in the cw quasi-resonant excitation scheme used for autocorrelation measurements, in that the resonant or near-resonant pumping of an exciton excited level may alter the second order autocorrelation function. In this latter case, the characteristic antibunching time could be driven by both the exciton radiative lifetime and the coherence time^77^. However, a quantitative assessment of this latter component would involve challenging experiments in the UV spectral range such as time-integrated four-wave mixing measurements in order to estimate the exact contribution of pure dephasing processes^78^. At this stage, we are still left with the fact that the *g*^(2)^(*τ*) decay time deduced at 300 K matches that of the long-lived component of the TRPL transients, *τ*_L_ (cf. Fig. 11). Unfortunately, this is in disagreement with the former attribution of *τ*_L_ to recombination originating from the low-energy exciton state, the RT *g*^(2)^(*τ*) trace being issued from the X_2_ line.

The reported thermal insensitivity of the long-lived component of TRPL transients raises an additional question about the temperature dependence of the emission features of GaN/AlN QDs. Indeed, thermal expansion of the dot and matrix materials is known to reduce their bandgap, and incidentally the QD emission energy. Hence, TRPL transients collected within a fixed energy window are supposed to originate from different QDs when increasing the temperature. We first estimated the energy shift by monitoring the change in the PL peak energy of the QD ensemble. We surprisingly observed a limited redshift of less than 10 meV, which diverges from the ∼60 meV redshift formerly reported on a GaN/AlN QD ensemble peaking around 4.4 eV^79^. On the other hand, we also had access via µ-PL measurements to the thermal shift experienced by QD_A_, which amounts to 60 meV. This value differs from the ∼90 meV redshift reported in previous works for single GaN QDs emitting at a similar energy^32,34^. Hence, it appears that instead of following a universal trend, the emission energy shift of each individual QD upon increasing temperature will likely depend on fluctuations, e.g., in their local strain field. Given the small variations in exciton lifetimes observed for high energy QDs (>3.8 eV), thermal energy shifts of tens of meV should not impact their recombination dynamics to a level that can be detected in our experiments. As far as QDs emitting below 3.8 eV are concerned, they display an increasing decay time, which roughly doubles every 200 meV as seen in Fig. 11. However, given the small thermal shift undergone by these large QDs, as can be verified once more from the marginal shift reported for the PL peak energy of the QD ensemble for dots emitting near 3.5 eV (cf. Fig. 7a), the related exciton lifetime variation upon increasing temperature is also expected to remain undetected.

### Review of excitonic decay in SA GaN/AlN QDs

**Fig. 12:**
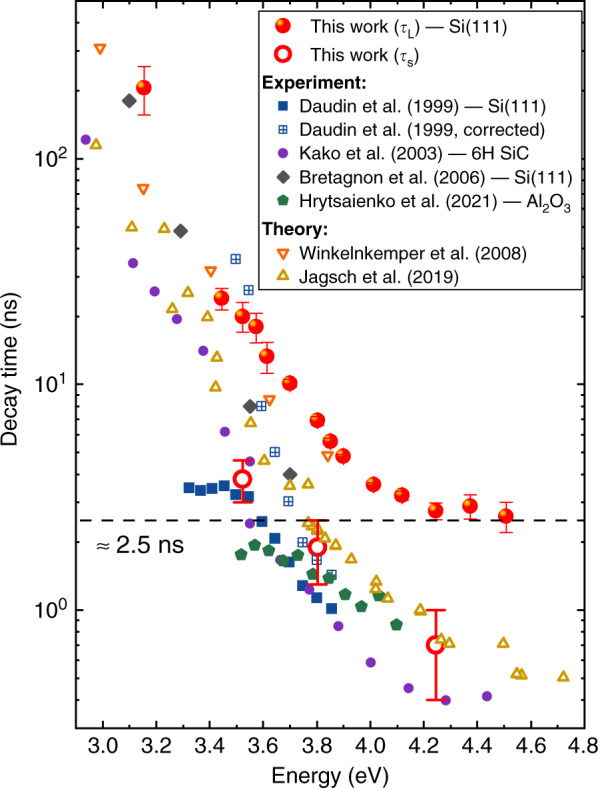
Comparison of exciton decay times reported in GaN/AlN QDs at cryogenic temperature. Experimental results^62–65^ are highlighted with filled markers and the substrate employed by each group is specified in the legend. Computed lifetimes (empty up and down triangles)^76,89^ were obtained for various aspect ratios and QD heights. Crossed squares correspond to experimental results after subtraction of a nonradiative recombination component, as detailed in ref. ^62^

To complete our analysis of the exciton recombination dynamics, we compare the long-lived decay times *τ*_L_ extracted at 5 K with experimental and theoretical exciton lifetimes published in the literature on SA GaN/AlN QDs^62–65^. In addition, the few short-lived decay times *τ*_S_ extracted from power-dependent TRPL experiments have also been added to the picture and all the results are shown in Fig. 12. The energy-dependent evolution of *τ*_L_ is in line with previous observations: an exponential decrease with decreasing dot size is observed for QDs emitting between 3 and 4.2 eV, followed by a saturation for higher energies. This trend is mainly driven by the electron-hole wave function overlap along the *c*-axis and can be satisfactorily reproduced by approximating the QDs as a two-dimensional quantum well subjected to a built-in electric field^65^. The same behavior is qualitatively observed for the short-lived component with *τ*_L_*/τ*_S_ ≃ 5. *τ*_L_ matches radiative lifetimes reported earlier on large QDs by Bretagnon et al.^64^. For small QDs, however, exciton lifetimes reported by Kako et al.^63^ and Hrytsaienko et al.^65^ are closer to the short lifetimes *τ*_S_. Let us note that these two groups additionally observed a longer-lived tail in the TRPL transients beyond exciton recombination, which resembles the bi-exponential decay we report here. These recombination processes were ascribed to QD refilling via interface related traps or free carriers present in the WL, and dark states acting as a reservoir. However, given the temperature-independence of *τ*_L_ in our case and the quasi-resonant excitation scheme we used, these hypotheses cannot account for our results. Besides, the long-lived decay we observe occurs on a too short timescale compared to that reported in ref. ^65^ and the long-lived component reported in this latter work does not change with emission energy, hence hinting at different mechanisms.

In essence, the data reported in Fig. 12 illustrate the variability in the exciton radiative recombination lifetime in SA GaN/AlN QDs. We can still distinguish between two QD excitation regimes: for large QDs, emitting below ∼3.5 eV, the in-plane electron-hole wave function confinement has a negligible impact on the QD PL properties. Hence, to a given QD height is associated a single QD energy–exciton lifetime combination. In other words, as for quantum wells, the QD properties are essentially dominated by the vertical confinement experienced by electron-hole pairs. For smaller QDs, on the other hand, lateral confinement is expected to significantly impact the QD emission energy^65^. However, fluctuations in the aspect ratio cannot solely account for exciton lifetimes differing by up to one order of magnitude for those emitting above 4 eV. Alternatively, the relief of tensile strain in the AlN matrix induced by microcracks has been shown to increase the excitonic lifetimes of SA GaN/AlN QDs grown on Si(111) substrates^80^. Thus, we reckon that the spread in experimental results is also likely caused by variations in the different strain field applied on QDs grown under distinct conditions. Despite the partial strain relaxation of the AlN matrix after growing a few monolayers, a residual stress originating from the substrate still remains. Thus, for our GaN QDs grown on Si(111) substrate the AlN matrix is still subjected to some biaxial tensile strain^80^. In this regard, we point out that the same substrate was used by Bretagnon et al.^64^ for the growth of their dots, whose measured exciton decay times are the closest to ours. Shorter lifetimes have been reported on samples grown on *c*-plane sapphire^62,65^ and 6H-SiC substrates^63^ for which the AlN matrix is largely relaxed^81,82^. Eventually, the QD optical properties will also depend on how the strain of the AlN matrix is impacting the GaN dot morphology (interatomic spacing, etc.). Our QDs are characterized by an ∼1.5 monolayer thick WL, about twice thinner than the one reported in earlier studies^62–65^. Hence, we can expect the AlN matrix to exert a stronger strain on our QDs than in other samples. Besides, a thinner WL should result in a stronger electronic confinement of the electrons and holes trapped in the QDs^83,84^, impacting both the exciton lifetime and the emission energy. In a pyramidal metal-polar GaN QD, however, the hole state is strongly confined due to its large effective mass and the electron is localized at the top of the dot. Hence, the impact of the WL thickness on their respective wave functions may be limited.

In summary, changes in the strain state from one type of sample to the other related to the nature of the substrates and the thickness of the WL could account for the reported variations in the exciton lifetime. In this regard, systematic micro-Raman spectroscopy measurements could be a complimentary and insightful technique to draw correlations between the strain state of QD ensembles and the measured lifetimes.

## Discussion

In conclusion, we have studied the evolution of the SPE behavior of polar SA GaN/AlN QDs from 5 K up to RT and provided a detailed description of the framework used to analyze their luminescence properties. We have reported single photon purity values as low as *g*^(2)^(0) = 0.05 ± 0.02 at 5 K and 0.17 ± 0.08 at 300 K by taking advantage of the large spectral separation between exciton and biexciton emission lines on the one hand and the quasi-resonant excitation scheme on the other hand. We have subsequently investigated the recombination dynamics of excitons for energies ranging from 3.2 to 4.5 eV, i.e., for QDs that emit both below and above the bulk GaN bandgap. This allowed us to evidence a two-component recombination process that persists in the low power density regime for any QD emission energies. Both fast and slow decay rate components are observed to drop significantly with increasing QD size. This follows the characteristic increase in exciton lifetime related to decreasing electron-hole wave function overlap along the vertical *c*-axis in polar quantum heterostructures. The long-lived exponential decay time remains constant, within experimental uncertainties, from cryogenic temperatures up to 300 K. This allowed us to associate it with a radiative recombination process stemming from an exciton bright state. This temperature invariance of the QD photoluminescence decay discards the contribution of a long-lived dark state reservoir to the bi-exponential dynamics, in contrast with earlier results obtained on CdSe QDs^66,67,85^. The latter result also confirms the negligible influence of nonradiative processes on the exciton recombination dynamics of GaN/AlN QDs, regardless of their size. As such, these QDs truly emerge as a robust platform for RT quantum applications, where the single photon purity of alternative epitaxial QD emitters breaks down^86^.

## Materials and Methods

**Fig. 13:**
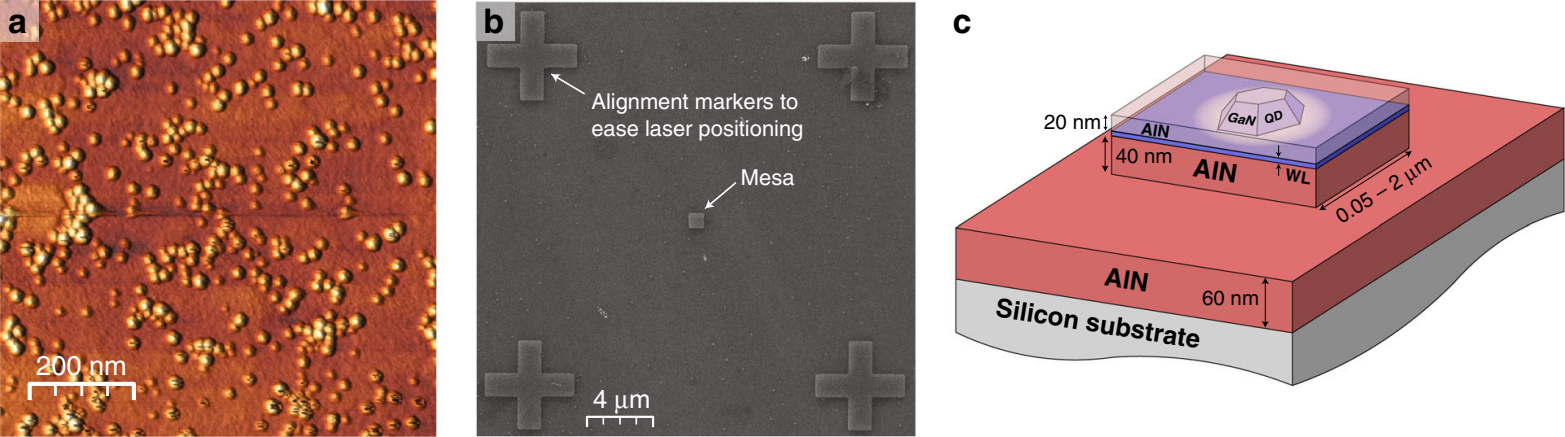
Description of the sample structure. **a** 1 × 1 µm^2^ AFM image taken in height mode of the top plane of GaN QDs displaying a broad distribution of dot sizes. **b** 26 × 26 µm^2^ secondary electron image of the SA QD sample after evaporation of the top QD plane and etching into mesa structures. The displayed mesa is 1 µm wide. **c** Sketch of the mesa structure. In practice, several QDs can be present on each mesa depending upon their dimension

The wurtzite SA QD sample under scrutiny was grown by ammonia-source molecular beam epitaxy (NH_3_-MBE). It first consists of a 100-nm-thick metal-polar AlN layer deposited on a Si(111) substrate. Then, a plane of SA GaN QDs (∼5-monolayer-thick) was grown and capped by a 20-nm-thick AlN barrier. At the surface, an additional uncapped plane of QDs was grown to check their size and density using atomic force microscopy (AFM) imaging (Fig. 13a). This latter QD plane was then evaporated under vacuum in the MBE chamber to guarantee the integrity of the remaining QD layer. The WL emission was detected by cathodoluminescence at 5.3 eV at a temperature of 12 K, which corresponds to a GaN WL thickness of ∼1.5 monolayers^87^. The sample was then patterned into mesas scaling from 0.05 × 0.05 µm^2^ up to 2 × 2 µm^2^ by electron beam lithography and subsequent etching down to the AlN buffer layer. Figure 13b presents a top view of the processed sample obtained by scanning electron microscopy and the mesa structure is schematized in Fig. 13c. Further details about SA QD growth can be found in Tamariz et al.^88^.

µ-PL and second order correlation function measurements were performed under quasi-resonant excitation by generating electron-hole pairs in the QD excited states. To this aim, we employed two different continuous wave (cw) laser light sources emitting either at 266 nm (4.66 eV) or at 244 nm (5.08 eV), i.e., below the WL transition energy. Excited states subsequently transit toward the optically active exciton ground states (X_1,2_) via phonon-assisted relaxation. We collected *g*^(2)^(*τ*) traces using an HBT interferometer with a time resolution of 220 ps. We recorded TRPL transients using a pulsed 266 nm laser with an 8.45 kHz repetition rate and a pulse duration of 440 ps, which is shorter than the GaN/AlN QD decay rate. The experimental setup is further detailed in the SI Sec. [S1].

## Supplementary information

Supplementary Information

## References

[1] Dowling JP, Milburn GJ (2003). Quantum technology: the second quantum revolution. Philos. Trans. R. Soc. A: Math. Phys. Eng. Sci..

[2] Aharonovich I, Englund D, Toth M (2016). Solid-state single-photon emitters. Nat. Photonics.

[3] Kurtsiefer C (2000). Stable solid-state source of single photons. Phys. Rev. Lett..

[4] Aharonovich I, Neu E (2014). Diamond nanophotonics. Adv. Opt. Mater..

[5] Bogdanov SI (2018). Ultrabright room-temperature sub-nanosecond emission from single nitrogen-vacancy centers coupled to nanopatch antennas. Nano Lett..

[6] He YM (2015). Single quantum emitters in monolayer semiconductors. Nat. Nanotechnol..

[7] Tran TT (2016). Quantum emission from hexagonal boron nitride monolayers. Nat. Nanotechnol..

[8] Berhane AM (2017). Bright room-temperature single-photon emission from defects in gallium nitride. Adv. Mater..

[9] Zhou Y (2018). Room temperature solid-state quantum emitters in the telecom range. Sci. Adv..

[10] Senellart P, Solomon G, White A (2017). High-performance semiconductor quantum-dot single-photon sources. Nat. Nanotechnol..

[11] Holmes MJ, Arita M, Arakawa Y (2019). III-nitride quantum dots as single photon emitters. Semicond. Sci. Technol..

[12] Arakawa Y, Holmes MJ (2020). Progress in quantum-dot single photon sources for quantum information technologies: a broad spectrum overview. Appl. Phys. Rev..

[13] Ostapenko IA (2012). Exciton acoustic-phonon coupling in single GaN/AlN quantum dots. Phys. Rev. B.

[14] Bardoux R (2006). Photoluminescence of single GaN/AlN hexagonal quantum dots on Si (111): spectral diffusion effects. Phys. Rev. B.

[15] Gao K (2017). Nanosecond-scale spectral diffusion in the single photon emission of a GaN quantum dot. AIP Adv..

[16] Arita M (2017). Ultraclean single photon emission from a GaN quantum dot. Nano Lett..

[17] Hönig G (2013). Identification of electric dipole moments of excitonic complexes in nitride-based quantum dots. Phys. Rev. B.

[18] Kindel C (2014). Spectral diffusion in nitride quantum dots: emission energy-dependent linewidths broadening via giant built-in dipole moments. Phys. Status Solidi RRL.

[19] Fonoberov VA, Balandin AA (2003). Excitonic properties of strained wurtzite and zinc-blende GaN/Al_*x*_Ga_1-*x*_N quantum dots. J. Appl. Phys..

[20] Sergent S (2013). Narrow spectral linewidth of single zinc-blende GaN/AlN self-assembled quantum dots. Appl. Phys. Lett..

[21] Wang X (2019). III-V compounds as single photon emitters. J. Semicond..

[22] Castelletto S (2020). Hexagonal boron nitride: a review of the emerging material platform for single-photon sources and the spin-photon interface. Beilstein J. Nanotechnol..

[23] Scarani V (2009). The security of practical quantum key distribution. Rev. Mod. Phys..

[24] Lo HK, Curty M, Tamaki K (2014). Secure quantum key distribution. Nat. Photon..

[25] White AG (1998). “Interaction-free” imaging. Phys. Rev. A.

[26] Cheung JY (2007). The quantum candela: a re-definition of the standard units for optical radiation. J. Mod. Opt..

[27] Deshpande S (2013). Electrically driven polarized single-photon emission from an InGaN quantum dot in a GaN nanowire. Nat. Commun..

[28] Damilano B (1999). From visible to white light emission by GaN quantum dots on Si(111) substrate. Appl. Phys. Lett..

[29] Frost, T. et al. InGaN/GaN quantum dot red (*λ* = 630 nm) Laser. *IEEE J. Quantum Electron.* **49**, 923 (2013).

[30] Verma J (2014). Tunnel-injection quantum dot deep-ultraviolet light-emitting diodes with polarization-induced doping in III-nitride heterostructures. Appl. Phys. Lett..

[31] Reilly CE (2019). Infrared luminescence from N-polar InN quantum dots and thin films grown by metal organic chemical vapor deposition. Appl. Phys. Lett..

[32] Holmes MJ (2014). Room-temperature triggered single photon emission from a III-nitride site-controlled nanowire quantum dot. Nano Lett..

[33] Holmes, M. et al. Single photons from a hot solid-state emitter at 350 K. *ACS Photonics* **3**, 543–546 (2016).

[34] Tamariz S (2020). Toward bright and pure single photon emitters at 300 K based on GaN quantum dots on silicon. ACS Photonics.

[35] Hönig G (2014). Manifestation of unconventional biexciton states in quantum dots. Nat. Commun..

[36] Arlery M (1999). Quantitative characterization of GaN quantum-dot structures in AlN by high-resolution transmission electron microscopy. Appl. Phys. Lett..

[37] Andreev AD, O’Reilly EP (2000). Theory of the electronic structure of GaN/AlN hexagonal quantum dots. Phys. Rev. B.

[38] Butté, R. & Grandjean, N. Effects of polarization in optoelectronic quantum structures. in Polarization Effects in Semiconductors: From Ab Initio Theory to Device Applications (eds Wood, C. & Jena, D.), 467–511 (Boston, MA: Springer, 2008).

[39] Ranjan V (2003). Self-consistent calculations of the optical properties of GaN quantum dots. Phys. Rev. B.

[40] Andreev AD, O’Reilly EP (2001). Optical transitions and radiative lifetime in GaN/AlN self-organized quantum dots. Appl. Phys. Lett..

[41] Schliwa, A., Hönig, G. & Bimberg, D. Electronic properties of III-V quantum dots. in Multi-Band Effective Mass Approximations: Advanced Mathematical Models and Numerical Techniques (eds Ehrhardt, M. & Koprucki, T.), 57–85 (Cham: Springer, 2014).

[42] Widmann F (1998). Blue-light emission from GaN self-assembled quantum dots due to giant piezoelectric effect. Phys. Rev. B.

[43] Hoshino K, Kako S, Arakawa Y (2004). Formation and optical properties of stacked GaN self-assembled quantum dots grown by metalorganic chemical vapor deposition. Appl. Phys. Lett..

[44] Rodina AV (2001). Free excitons in wurtzite GaN. Phys. Rev. B.

[45] Ivchenko EL (1997). Fine structure of excitonic levels in semiconductor nanostructures. Phys. Status Solidi A.

[46] Kindel C (2010). Exciton fine-structure splitting in GaN/AlN quantum dots. Phys. Rev. B.

[47] Seguin R (2006). Quantum-dot size dependence of exciton fine-structure splitting. Phys. E: Low.-Dimens. Syst. Nanostruct..

[48] Liu F (2013). Spin dynamics of negatively charged excitons in CdSe/CdS colloidal nanocrystals. Phys. Rev. B.

[49] Regelman DV (2001). Semiconductor quantum dot: a quantum light source of multicolor photons with tunable statistics. Phys. Rev. Lett..

[50] Mizrahi U (2003). Non-classical light generated by a quantum dot: multi-color photons with tunable statistics. Synth. Met..

[51] Dekel E (2000). Carrier-carrier correlations in an optically excited single semiconductor quantum dot. Phys. Rev. B.

[52] Kindel, C. Study on optical polarization in hexagonal gallium nitride quantum dots. PhD Thesis, University of Tokyo (2010).

[53] Sun, X. et al. Excitation and emission dynamics of a single photon emitting InGaN quantum dot in a photonic horn structure. *Superlattices Microstruct.* **145**, 106575 (2020).

[54] Ohnesorge B (1996). Rapid carrier relaxation in self-assembled In_*x*_Ga_1-*x*_As/GaAs quantum dots. Phys. Rev. B.

[55] Ferreira, R. & Bastard, G. Phonon-assisted capture and intradot Auger relaxation in quantum dots. *Appl. Phys. Lett.* **74**, 2818–2820 (1999).

[56] Gao K (2019). Spectral diffusion time scales in InGaN/GaN quantum dots. Appl. Phys. Lett..

[57] Holmes MJ (2021). Pure single-photon emission from an InGaN/GaN quantum dot. APL Mater..

[58] Sallen G (2010). Subnanosecond spectral diffusion measurement using photon correlation. Nat. Photonics.

[59] Fischer AJ (1997). Femtosecond four-wave-mixing studies of nearly homogeneously broadened excitons in GaN. Phys. Rev. B.

[60] Carmele A, Knorr A (2011). Analytical solution of the quantum-state tomography of the biexciton cascade in semiconductor quantum dots: pure dephasing does not affect entanglement. Phys. Rev. B.

[61] Callsen G (2013). Steering photon statistics in single quantum dots: from one-to two-photon emission. Phys. Rev. B.

[62] Daudin B (1999). Piezoelectric properties of GaN self-organized quantum dots. MRS Internet J. Nitride Semicond. Res..

[63] Kako S (2003). Size-dependent radiative decay time of excitons in GaN/AlN self-assembled quantum dots. Appl. Phys. Lett..

[64] Bretagnon T (2006). Radiative lifetime of a single electron-hole pair in GaN/AlN quantum dots. Phys. Rev. B.

[65] Hrytsaienko M (2021). Dark-level trapping, lateral confinement, and built-in electric field contributions to the carrier dynamics in *c*-plane GaN/AlN quantum dots emitting in the UV range. J. Appl. Phys..

[66] Labeau O, Tamarat P, Lounis B (2003). Temperature dependence of the luminescence lifetime of single CdSe/ZnS quantum dots. Phys. Rev. Lett..

[67] Sallen G (2009). Exciton dynamics of a single quantum dot embedded in a nanowire. Phys. Rev. B.

[68] Le Roux F (2017). Temperature dependence of the single photon emission from interface-fluctuation GaN quantum dots. Sci. Rep..

[69] Brouri R (2000). Photon antibunching in the fluorescence of individual color centers in diamond. Opt. Lett..

[70] Tomić S, Vukmirović N (2009). Excitonic and biexcitonic properties of single GaN quantum dots modeled by 8-band k⋅p theory and configuration-interaction method. Phys. Rev. B.

[71] Melnychuk C, Guyot-Sionnest P (2021). Multicarrier dynamics in quantum dots. Chem. Rev..

[72] Kurzmann A (2016). Auger recombination in self-assembled quantum dots: quenching and broadening of the charged exciton transition. Nano Lett..

[73] Santori C (2002). Time-resolved spectroscopy of multiexcitonic decay in an InAs quantum dot. Phys. Rev. B.

[74] Lasher G, Stern F (1964). Spontaneous and stimulated recombination radiation in semiconductors. Phys. Rev..

[75] Renard J (2009). Suppression of nonradiative processes in long-lived polar GaN/AlN quantum dots. Appl. Phys. Lett..

[76] Winkelnkemper M (2008). GaN/AlN quantum dots for single qubit emitters. J. Phys.: Condens. Matter.

[77] Nguyen HS (2011). Ultra-coherent single photon source. Appl. Phys. Lett..

[78] Borri P (2001). Ultralong dephasing time in InGaAs quantum dots. Phys. Rev. Lett..

[79] Renard J (2009). Evidence for quantum-confined Stark effect in GaN/AlN quantum dots in nanowires. Phys. Rev. B.

[80] Sarusi G (2007). Microcrack-induced strain relief in GaN/AlN quantum dots grown on Si(111). Phys. Rev. B.

[81] Ponce FA (1994). Crystalline structure of AlGaN epitaxy on sapphire using AlN buffer layers. Appl. Phys. Lett..

[82] Ponce FA, Krusor BS (1995). Microstructure of GaN epitaxy on SiC using AlN buffer layers. Appl. Phys. Lett..

[83] Sanguinetti S (2003). Modified droplet epitaxy GaAs/AlGaAs quantum dots grown on a variable thickness wetting layer. J. Cryst. Growth.

[84] Sun C (2012). Wetting layers effect on InAs/GaAs quantum dots. Phys. B: Condens. Matter.

[85] Patton B, Langbein W, Woggon U (2003). Trion, biexciton, and exciton dynamics in single self-assembled CdSe quantum dots. Phys. Rev. B.

[86] Yu P (2019). Nanowire quantum dot surface engineering for high temperature single photon emission. ACS Nano.

[87] Liu C (2018). 234 nm and 246 nm AlN-Delta-GaN quantum well deep ultraviolet light-emitting diodes. Appl. Phys. Lett..

[88] Tamariz S, Callsen G, Grandjean N (2019). Density control of GaN quantum dots on AlN single crystal. Appl. Phys. Lett..

[89] Jagsch ST (2019). Ground-state resonant two-photon transitions in wurtzite GaN/AlN quantum dots. Phys. Rev. B.

