# Supplementary material for: Single photon emission and recombination dynamics in self-assembled GaN/AlN quantum dots
Source: Light Sci Appl. 2022 Apr 28;11:114. doi: 10.1038/s41377-022-00799-4 (PMC9046275; doi:10.1038/s41377-022-00799-4)
Supplement: Supplementary file 1 — Supplementary Information [file 41377_2022_799_MOESM1_ESM.pdf]

Supplementary Information for: Single photon emission and recombination dynamics in self-assembled GaN/AlN quantum dots

Johann Stachurski,[1,*] Sebastian Tamariz,[1,2] Gordon Callsen,[1,3] Raphaël Butté,[1] and Nicolas Grandjean[1]

[1]*Institute of Physics, École Polytechnique Fédérale de Lausanne, EPFL, CH-1015 Lausanne, Switzerland*

[2]*Current address: Université Côte d'Azur, CNRS, CRHEA, F-06560 Valbonne, France*

[3]*Current address: Institut für Festkörperphysik, Universität Bremen, 28359 Bremen, Germany*

[*] johann.stachurski@epfl.ch

S1. µ-PL AND TRPL OPTICAL CHARACTERIZATION METHODS

Microphotoluminescence (µ-PL) and second order auto-correlation ($g^{(2)}(\tau)$) measurements were performed under continuous-wave (cw) excitation using either a 266 nm (4.66 eV) Nd:YAG laser from Crylas or a 488 nm solid state laser (Coherent Genesis CX SLM) frequency-doubled to 244 nm (5.08 eV) by a Spectra-Physics WaveTrain. For time-resolved photoluminescence (TRPL) measurements, we used a Teem Photonics Microchip 266 nm pulsed Nd:YAG laser with a repetition rate of 8.45 kHz and a pulse duration of 440 ps. The different sources are depicted on the illustration of the setup shown in Fig. S1. Under cw excitation, we focused the laser beam with a ×80 Mitutoyo microscope objective (NA = 0.55) to reach a spot size of ≃ 1 µm (Fig. S2).

For TRPL measurements, the laser was focused using a conventional plano-convex lens of 2.5 cm focal length to reach a spot size of ≃ 100 µm. In order to optimize the collection of the sample photoluminescence, we guided each laser beam using a 90:10 transmission/reflection beam sampler onto the sample, which was placed in a closed-cycle helium cryostat (Cryostation C2 from Montana Instruments, Inc.) to cool it down to 5 K. µ-PL mapping of the

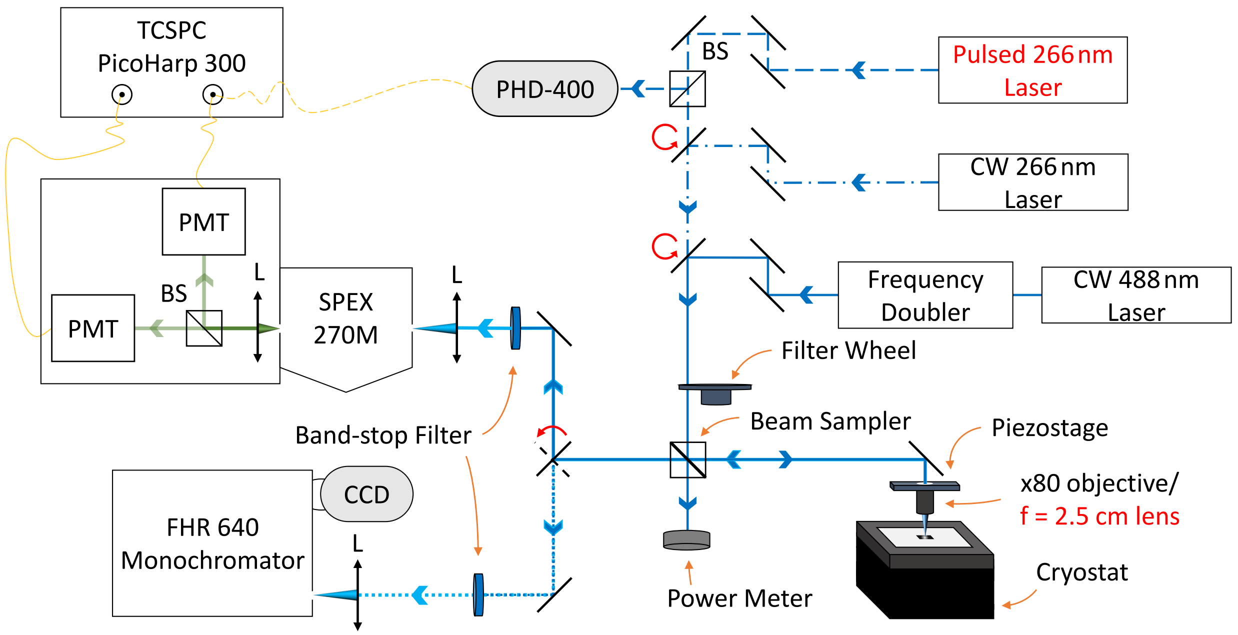

FIG. S1. Schematic illustration of the optical setup. BS and L stand for beam splitter and lens, respectively. Dashed, dotted and dash-dotted lines correspond to alternative optical paths. The red arrows indicate flippable mirrors.

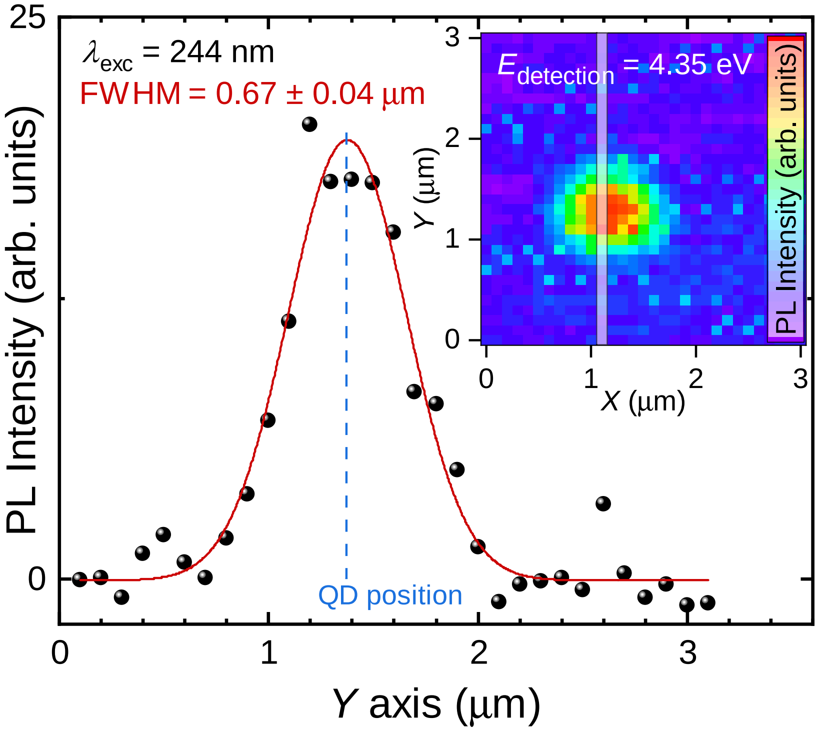

FIG. S2. Gaussian profile of the µ-PL 244 nm laser spot recorded by scanning the PL emission of a single QD emitting at $E = 4.35\,\text{eV}$. Inset: mapscan centered on the QD. The PL profile corresponds to the points encompassed into the white stripe. The laser spot displays a Gaussian profile with a full width at half-maximum (FWHM) of 0.67 µm.

surface was performed by mounting the microscope objective on a Physik Instrumente P-612.2 XY piezostage. µ-PL spectra were recorded with a Horiba Symphony II UV-enhanced charge-coupled device (CCD) coupled to a Horiba FHR 640 monochromator equipped with 1800 $\text{l}\cdot\text{mm}^{-1}$ and 150 $\text{l}\cdot\text{mm}^{-1}$ holographic gratings. Autocorrelation measurements were performed using a Hanbury-Brown and Twiss (HBT) interferometer with two PicoQuant PMA 175 photomultiplier tubes linked to a PicoHarp 300 time-correlated single photon counter (TCSPC). For TPRL measurements, a PHD-400 photodiode module was alternatively connected to one channel of the TCSPC to use the laser pulses as a trigger. We measured a time resolution of 220 ps for the autocorrelation setup. The bandpass for $g^{(2)}$ and TRPL measurements was selected using a SPEX 270M monochromator with a 2400 $\text{l}\cdot\text{mm}^{-1}$ grating and a slit opening of 1.6 mm. The collection efficiency of the HBT setup was estimated to $(0.27 \pm 0.10)\,\%$. Further details can be found in Tamariz *et al.* [1].

S2. PHOTON EXTRACTION EFFICIENCY

Besides the HBT setup collection efficiency reported above, we should ideally compute the photon extraction efficiency from our sample to relate the detection rate to the real QD emission rate. While an exact computation of this latter efficiency goes beyond the scope of this work given the complexity introduced by the mesa geometry, we can nonetheless give a lower bound value for the extraction efficiency $\eta_{\mathrm{ext,min}}$ by approximating our system to a conventional planar geometry while only considering photons emitted toward the top AlN/air interface, i.e., the photon flux partly reflected at the AlN/silicon substrate interface is neglected. Within this framework, the QD is considered as an isotropic emitter and the optical losses are only assumed to occur at the AlN/air interface. Owing to its wide bandgap, the AlN layer is fully transparent in the whole domain of emission of the QDs. The transmission coefficient T under normal incidence, also known as the dielectric efficiency [2], obtained using the refractive index of AlN $n_{\mathrm{AlN}} \approx 2.13$ [3] around 4.5 eV, is defined as:

$$T = 1 - \frac{(n_{\mathrm{AlN}} - 1)^2}{(n_{\mathrm{AlN}} + 1)^2} = 0.87. \tag{S1}$$

$\eta_{\mathrm{ext,min}}$ depends on the solid angle leading to light extraction Ω_c, which simply writes:

$$\Omega_c = \frac{1}{2}(1 - \cos(\theta_c)) \cdot \Omega_{tot}, \tag{S2}$$

where $\theta_c = \arcsin\left(n_{\mathrm{AlN}}^{-1}\right)$ is the critical angle leading to total internal reflection and $\Omega_{\mathrm{tot}} = 4\pi$ is the total solid angle. As a result, we get $\eta_{\mathrm{ext,min}} \approx T \cdot \Omega_c/\Omega_{\mathrm{tot}}$, which leads to $\eta_{\mathrm{ext,min}} = 5.1\,\%$. In pratice, we expect the actual extraction efficiency to be significantly higher. Note also that given the much smaller refractive index value of wide bandgap semiconductors compared to their III-arsenide counterparts, the present $\eta_{\mathrm{ext,min}}$ value is more than three times larger than that we would obtain for InAs/GaAs QDs.

S3. POLARIZATION- AND TEMPERATURE-DEPENDENT QD PHOTOLUMINESCENCE

Here, we present additional photoluminescence measurements, mostly performed on the quantum dot (QD) labelled $\mathrm{QD_A}$. This QD is especially interesting for its brightness and the large spectral separation between its emission lines. Additional peaks are however observed in the same spectral window and are attributed to another QD based on their different linear

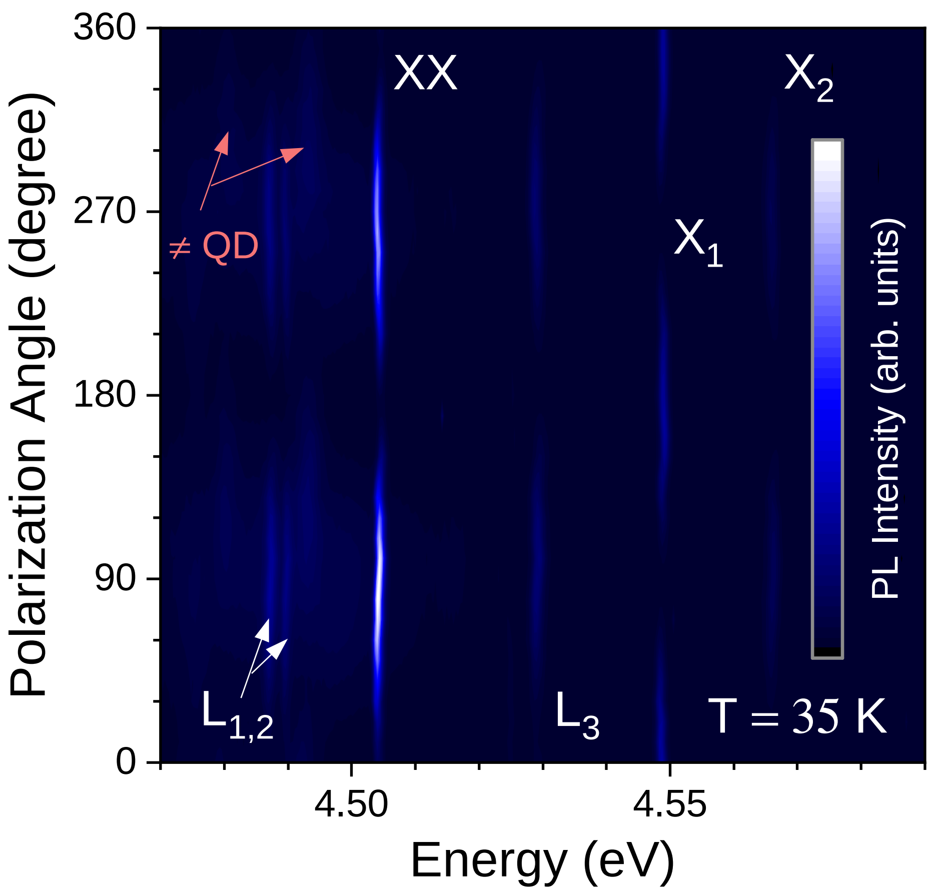

FIG. S3. Color map of polarization-dependent photoluminescence spectra of QD_A recorded at $T =$ 35 K. The lines L_1 to L_3 have not been clearly identified yet. The low energy lines are overlapping with the emission of another QD. Data taken under cw excitation at a wavelength of 244 nm and a power density of $400\,kW\,cm^{-2}$.

polarization orientation (Fig. S3). The two QD_A biexciton (XX) lines are not resolved, while the two exciton lines ($X_{1,2}$) are fully separated. The lines L_1 to L_3 have not been assigned so far, but they show the same linear polarization orientation than XX and X_2. X_1 is cross-polarized to all other lines of QD_A. Apart from L_3, similar patterns have been observed earlier on GaN/AlN QDs [1, 4, 5].

The comparison between the polarization-dependent photoluminescence measurements of different QDs performed at cryogenic temperature suggests that the polarization of exciton emission lines could be pinned by crystallographic axes. As an illustration, Fig. S4 shows the polar representation of the normalized intensity of three X_1 exciton emission lines. The

data are well reproduced when using the relationship [6]:

$$I(\theta) = a + b \cdot \cos(\theta - \theta_0)^2, \tag{S3}$$

where a, b and θ_0 are fitting parameters. The linear polarization degree $\rho = (I_{\text{max}} - I_{\text{min}})/(I_{\text{max}} + I_{\text{min}})$, where I_{max} and I_{min} are the maximum and minimum X_1 line integrated μ−PL intensity, respectively, is systematically above 95 %. The polarization axis of the QD_A-X_1 line is tilted by about 60° with respect to $QD_{B,C}$-X_1, hence recalling the in-plane hexagonal symmetry of wurtzite GaN. However, we do not have a statistically significant set of data to fully validate this preliminary result. A previous report on the polarized emission of single GaN/AlN QDs by Bardoux and co-workers [6] did not support the existence of such preferential polarization axes while no unequivocal conclusion arose from the results reported in the PhD thesis by Kindel [7].

At 5 K, XX and X_1 dominate the emission spectrum. The transition from dark to bright states requires the simultaneous absorption of phonons which match their energy difference [4]. Assuming a negligible dark-state splitting, the intensity of X_2 increases when the thermal energy $k_B T$ approaches $E_{\text{DB}} + E_{\text{BB}}/2$, where E_{BB} (E_{DB}) is the bright (dark-to-bright) state splitting. As observed in Fig. S5, X_2 takes over X_1 in intensity around 40 K.

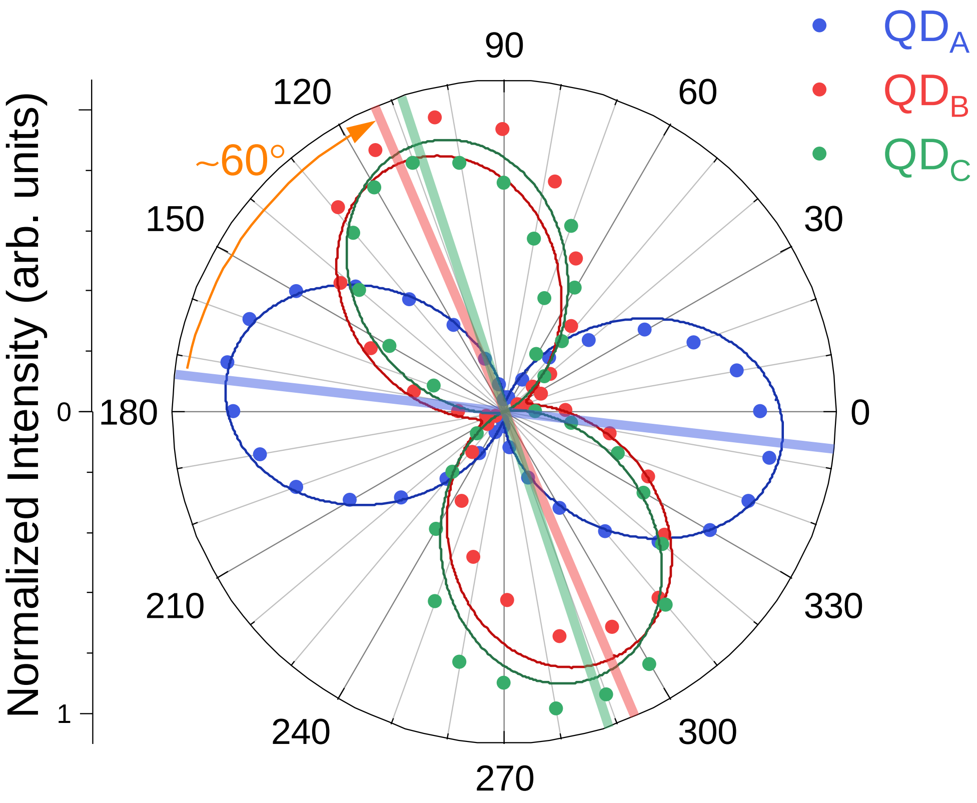

FIG. S4. Polar representation of the normalized intensity of the X_1 line measured for three different QDs. Solid lines are fitted using Eq. (S3). All excitonic lines display a strong degree of linear polarization ($\rho > 0.95$) with polarization axes highlighted with thick solid lines on the figure. Polarization axes of QD_A-X_1 and QD_A-X_2 are rotated by $(61 \pm 2)^\circ$.

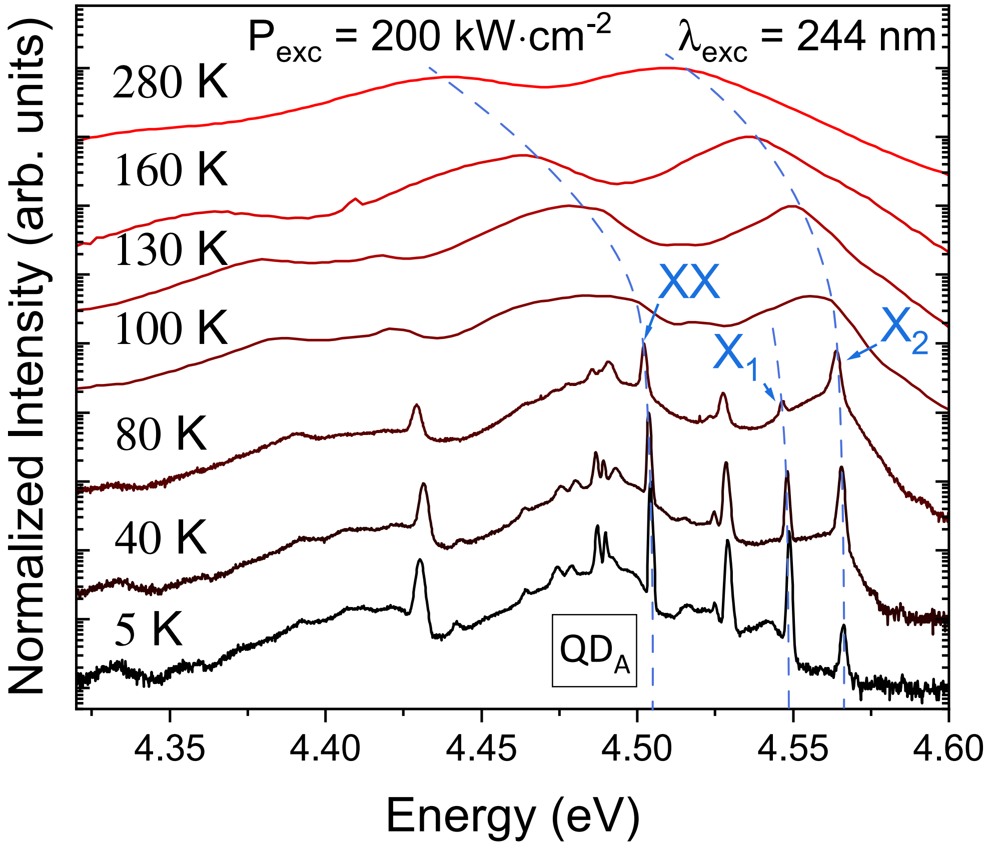

FIG. S5. Temperature-dependent µ-PL spectra recorded on QD_A. The exciton and biexciton lines are labelled. The dashed lines serve as a guide to the eye. X_2 takes over X_1 around 40 K. The low energy lines are overlapping with the emission of another QD. The smoothening observed between 80 K and 100 K spectra stems from a grating change. Low (up to $T = 80$ K) and high temperature spectra were recorded with a 1800 l·mm^{-1} and a 150 l·mm^{-1} grating, respectively.

S4. LIMITATIONS OF THE SECOND ORDER AUTO-CORRELATION FITTING FUNCTION AND IMPACT OF CHARGE FLUCTUATIONS

The standard multi-level model (Eq. 1 of the main text) used to fit the second order autocorrelation function measurements predicts a narrowing of the antibunching dip as a consequence of a bunching behavior. This arises from an increase in the QD mean occupation number $\mu = \Pi/\gamma$, where the decay rate $\gamma = 1/\tau_{\mathrm{decay}}$, with τ_{decay} the exciton decay time, is assumed fixed and the pump rate Π depends on the excitation power density. This narrowing is visible in Fig. 2**c** of the main text. However, the fit partially diverges from the data when γ is kept constant. A more consistent fit can only be obtained when assuming an increase in γ with excitation power density. Such a fit yields a τ_{decay} value that drops from about 19 to 13 ns between 40 and 400 kW cm^{-2}. This variation may be accounted for by different phenomena that add to the contribution of dark states to the exciton dynamics discussed in the main text. These alternative explanations are discussed hereafter.

In polar III-nitride QDs, the strong internal electric field $\mathbf{E}_{\mathrm{in}}$ due to the macroscopic polarization mismatch between the dot and the barrier materials is responsible for the out-of-plane separation of electron and hole wave functions. This separation modifies the oscillator strength of the exciton ground state, which in turn drives the variations in the decay rate with QD size. A variation in $\mathbf{E}_{\mathrm{in}}$ with increasing excitation power density could therefore possibly explain the change in γ observed via the correlation measurements shown in Fig. 2**c** of the main text. Screening of the built-in field due to trapping of additional carriers at high excitation power densities has already been reported in GaN/AlN QDs [8] and may, at first sight, explain this variation. At the level of a single QD, however, this results in sharp changes in the QD emission energy and in the observation of additional emission lines (biexciton, charged exciton, etc.), which do not contribute to the $g^{(2)}(\tau)$ traces. Hence, the fluctuations of $\mathbf{E}_{\mathrm{in}}$ can only be accounted for by charge fluctuations external to the QDs. A common way to picture it consists in considering the charge and discharge of defect states (DSs), whose origin could be ascribed to the presence of vacancies at the GaN/AlN interface, surface states, or point defects surrounding the QD. First, setting aside their exact origin, DSs are responsible for a time-dependent extrinsic electric field $\mathbf{E}_{\mathrm{ext}}(t)$, which creates an additional Stark shift of the exciton emission energy given by

$$\Delta E(t) = \boldsymbol{\mu} \cdot \mathbf{E}_{\mathrm{ext}}(t), \tag{S4}$$

where $\boldsymbol{\mu}$ is the exciton dipole moment, which is aligned along the c-axis and is oriented toward the top of the QD in metal-polar III-nitride QDs. DSs can be modelled as traps with given capture $\tau_{\downarrow}$ and escape $\tau_{\uparrow}$ times. At cryogenic temperature and under low excitation conditions, the phonon-mediated escape of trapped carriers is blocked and DSs are assumed to be mostly charged [9, 10]. Carrier escape is triggered when the sample is pumped and the higher the excitation power density, the lower the probability for a defect to be occupied. In the extreme situation where all the defects are depleted, the exciton energy gets ultimately shifted by an energy ΔE_{max}, which accounts for the contribution of all DSs. As developed in Ref. [7], for DSs in the close vicinity of polar III-nitride QDs, the exciton peaks are expected to shift with increasing excitation power density following

$$E(P_{\text{exc}}) = E_0 + \frac{\Delta E_{\text{max}}}{\sqrt{P_{\text{exc}}/P_{50\%}} + 1}. \tag{S5}$$

In Eq. (S5), E_0 corresponds to the emission energy of the excitonic line of interest when all DSs are filled whereas $P_{50\%}$ corresponds to the excitation power density for which the occupation probability of the DSs is equal to one half. The sign of ΔE_{max} depends on the overall positioning of the defects around the QDs and on the sign of the trapped carriers.

Such a shift has been consistently observed for various QDs, including QD_A on which most of the $g^{(2)}(\tau)$ traces considered in this work have been recorded. The corresponding results are thus reported for three different QDs in Fig. S6**a**. All of the investigated exciton emission lines displayed a redshift of about 0.5 to 2 meV. Interestingly, when adopting a linear excitation power density scale to show the same data (Fig. S6**b**), it is clearly seen that this shift mainly occurs for excitation power densities below 30 kW cm^{-2}. The steady redshift hints at a common origin for $\mathbf{E}_{\text{ext}}$, such as DSs located at the surface of the 20 nm thick AlN capping layer. An accumulation of point defects occurring above or below the GaN wetting layer (WL) could also account for this electric field of extrinsic origin. In this regard, Si impurities diffusing from the Si(111) substrate to the AlN buffer layer, even in minute concentration, or n-type O or C impurities are the most likely to be found in our sample as well as hydrogen-related charged defects.

Importantly enough, the observed shifts remain rather small ($\sim$1 meV, i.e., on the order of the FWHM of the $\text{X}_{1,2}$ emission lines), which contrasts with the 30 % reduction in τ_{decay} extracted from second order autocorrelation measurements when fitting each $g^{(2)}(\tau)$ trace

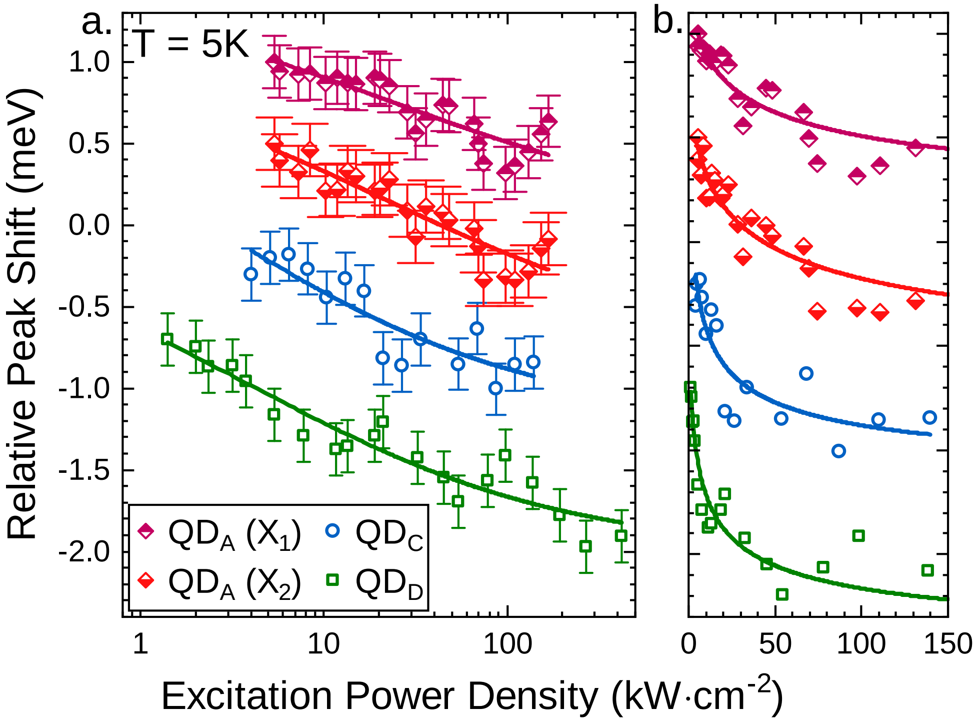

FIG. S6. Power-dependent evolution of the exciton peak energy for QDs emitting between 4.4 and 4.6 eV, recorded at 5 K and displayed in **a.** and **b.** using logarithmic and linear excitation power density scales, respectively. The results (data points) are fitted using Eq. S5 (solid lines). $QD_{A,C}$ were excited using the 244 nm laser line and QD_D using the 266 nm laser line. The shift reported for $QD_{C,D}$ is related to the X_1 line. An arbitrary energy offset was applied to each dataset for the sake of visual convenience. The magnitude of the error bars is related to the spectral separation between two consecutive pixels since our measurements are limited by the pixel size in the CCD.

individually. At first sight, such a decrease in τ_{decay} would be commensurate with a noticeable change in the oscillator strength of the exciton ground state and hence a large spectral shift. Indeed, in the energy range associated to these dots –$QD_{A,C,D}$ all emit above 4 eV– the energy shift leading to a change of 30 % in τ_{decay} would amount to hundreds of meV (cf. Fig. 11 of the main text). Thus, any screening of the built-in electric field can be excluded to explain the measured variation in the decay time. Since the amplitude of the electric field generated by DSs is on the order of a few MV m^{-1} [11], it cannot impact the built-in electric field of about 7-9 MV cm^{-1} [8, 12] significantly enough to explain the measured reduction in the decay time.

Besides variations in the built-in electric field experienced by the ground state exciton,

another reason for explaining the divergence between the fit with fixed γ and experimental $g^{(2)}(\tau)$ data can also be found in the original hypothesis used to develop the multi-level model. Indeed, to allow for a closed analytic form of the second order autocorrelation function in the framework of this model, the recombination rate of an N-exciton is assumed to be proportional to the number of electron-hole pairs in the QD, i.e., $\gamma_N = N \cdot \gamma$ [7]. As the excitation power density is increased and the bunching induced by a higher QD mean occupation number is strengthened, this approximation may become more questionable.

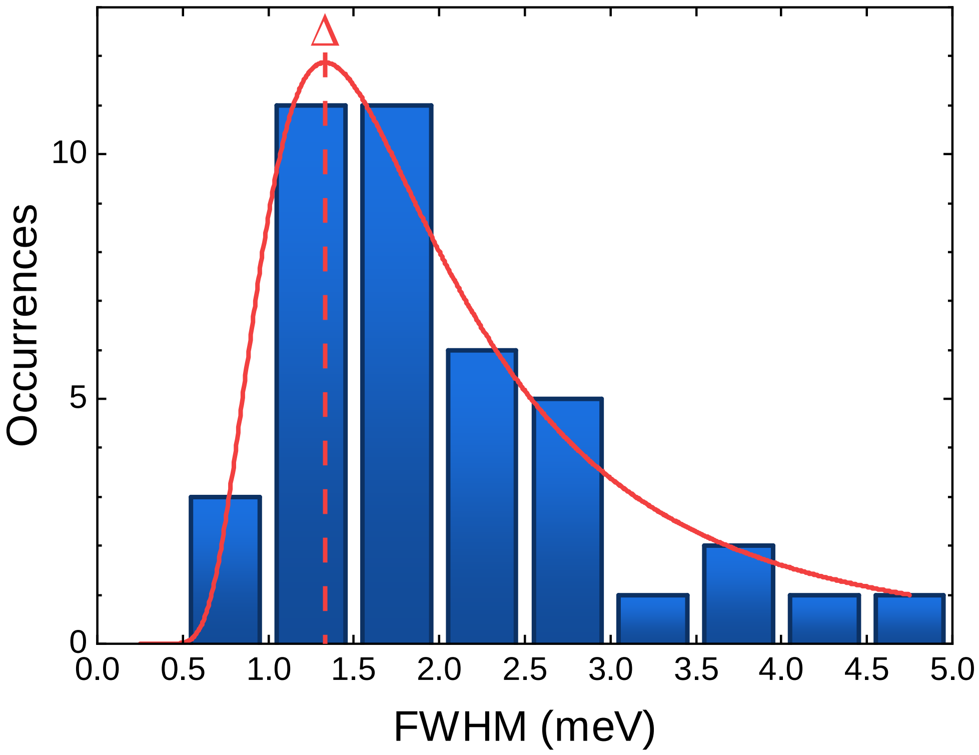

FIG. S7. Histogram of the excitonic peak linewidth distribution for QDs emitting between 4.65 and 4.85 eV. The linewidth distribution is fitted with a Fréchet function (Eq. S6) times a normalization parameter. The linewidth leading to the maximum value of the distribution, $\Delta = (1.3 \pm 0.3)$ meV, has been determined as an average over various binning conditions.

S5. ESTIMATION OF THE DEFECT DENSITY THROUGH QUANTUM DOT EMISSION LINEWIDTH STATISTICS

As shown in Ref. [7], spectral diffusion in III-nitride polar QDs is also responsible for a very specific excitonic linewidth distribution, which is highlighted in Fig. 1**d** of the main text. As detailed beforehand, QDs are impacted by the charging and discharging of neighboring DSs. While these charge fluctuations contribute to the broadening of exciton emission lines at cryogenic temperature, way beyond the natural linewidth, this feature can also be used to estimate the defect density in the dot surrounding.

To this end, we relied on the probability distribution function of the exciton linewidth for a given emission energy (E) that can be approached by a Fréchet distribution [7]:

$$f_{Fr}(E) = \frac{\alpha}{s} \cdot \left(\frac{E}{s}\right)^{-1-\alpha} \cdot \exp\left[-\left(\frac{E}{s}\right)^{-\alpha}\right], \tag{S6}$$

where the scaling parameter s and the shape parameter α are considered as free parameters here. The maximum value of $f_{Fr}(E)$ is associated to a linewidth value (Δ) that can then be used to estimate the point defect density $\tilde{\rho}$ using [7]:

$$\tilde{\rho} = \frac{3}{4\pi}\left[\frac{1}{2\sqrt{2\ln 2}}\frac{1}{\sqrt{p(1-p)}}\right]^{3/2}\left(\frac{\Delta}{\kappa e}\right)^{3/2}, \tag{S7}$$

where p is the occupation probability of a defect, e is the elementary charge, and κ is a numerically estimated coefficient that depends on the QD emission energy. Using $p = 1/2$ we can estimate a minimum value for $\tilde{\rho}$. Ideally, Δ is determined experimentally for a fixed emission energy by collecting the FWHM histogram of exciton emission lines within a narrow enough spectral range and by fitting the results with Eq. S6. Depending on the amount of recorded emission lines, this spectral range can be expanded to get a statistically significant dataset. In this work, we extracted the FWHM of all spectrally resolved exciton emission lines following a $100 \times 100\,\mu\mathrm{m}^2$ PL mapscan collected over a sample area processed into mesa structures. Results are displayed in Fig. 1**d** of the main text. From this set of measurements, we retained QDs emitting within a 200 meV interval centered at 4.75 eV, which led to the histogram shown in Fig. S7. While the high energy tail of the distribution is not ideally resolved, the number of occurrences remains large enough to estimate the position of the maximum of the distribution, namely $\Delta = (1.3 \pm 0.3)$ meV. The resulting $\tilde{\rho}$ value, $\sim 1 \times 10^{18}\,\mathrm{cm}^{-3}$, is a rough estimate of the minimum DS density located in the

vicinity of the present polar SA GaN/AlN QDs. In this respect, our value compares well to the $\sim 2 \times 10^{19}\,\mathrm{cm}^{-3}$ defect density computed in Ref. [7] for GaN/AlN QDs grown on SiC.

S6. IMPACT OF THE DETECTION WINDOW EXTENT ON THE SINGLE-PHOTON PURITY RECORDED BY SECOND ORDER AUTOCORRELATION MEASUREMENTS.

By taking advantage of the quasi-resonant excitation scheme adopted in this work, we have been able to record $g^{(2)}(\tau)$ traces with limited background emission at cryogenic temperature. Yet, as temperature increases, the thermal broadening of QD emission lines leads to an increased contribution of biexciton PL to the correlated counts and a reduction in the single-photon purity that follows accordingly. Thanks to the large biexciton binding energy of QDs emitting around 4.5 eV, single-photon emission could still be observed at RT, as described in the main text. We showed additionally that the resulting increase in $g^{(2)}(0)$ can be accounted for by considering the biexciton luminescence as an uncorrelated background [13]:

$$g^{(2)}(0) = 1 - \rho^2 \quad ; \quad \rho = \frac{I_X}{I_X + I_{XX}}, \tag{S8}$$

where I_X (I_{XX}) corresponds to the PL intensity of the exciton line (biexciton line) detected in the bandpass of the HBT interferometer. Both intensity terms can be determined by fitting the QD emission lines with separate peak functions (see inset of Fig. S8) and by integrating each component in the energy interval of the HBT setup. At 300 K and for an excitation power density of $400\,\mathrm{kW\,cm^{-2}}$, the single-photon purity of about $g^{(2)}(0) = 0.27$ estimated for $\mathrm{QD_A}$ using Eq. S8 (see Fig. S8) is very close to the value $g^{(2)}_{fit}(0) = (0.3 \pm 0.1)$ extracted from the second order autocorrelation function (see Fig. 4 in the main text). To strengthen this result, Eq. S8 should ideally be tested for different bandpasses. Our setup is unfortunately limited to a bandpass of 8 meV. Nevertheless, we can still infer the prospective evolution of the single-photon purity when including an increase in the spectral collection efficiency. Figure S8 highlights the prediction we made based on Eq. S8 and the exciton and biexciton intensities extracted by fitting the PL peaks. Thus, RT single-photon emission is foreseen to be maintained when collecting up to 90 % of the exciton PL. In other words, beyond such an exciton PL collection efficiency, the spurious contribution of the biexciton line in the bandpass leads to $g^{(2)}(0) \geq 0.5$. Besides, we should point out the remarkable stability

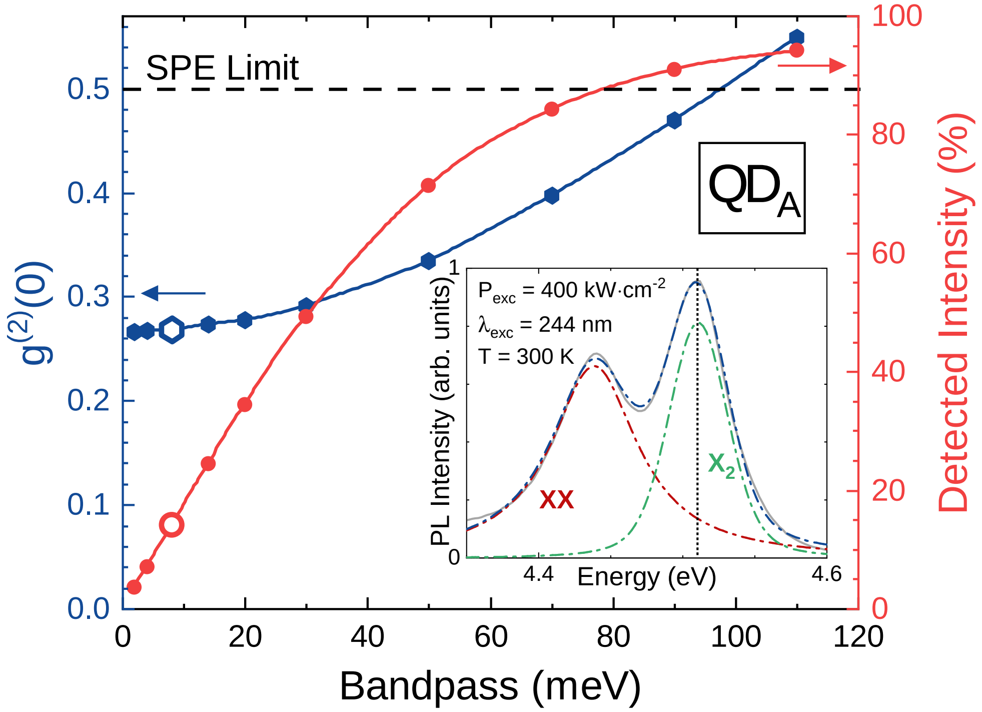

FIG. S8. Evolution of single-photon purity and collected exciton PL intensity as a function of the bandpass of the detection window. The detected PL intensity of the exciton line is given as a percentage of the total exciton intensity. The empty hexagon and dot underscore the 8 meV bandpass, which was used experimentally. The horizontal black dashed line outlines the 0.5 single-photon emission limit. Solid lines serve as a guide to the eye and all of the data points were estimated after the two-peak fitting of the QD PL, as depicted in the inset. The blue dashed line is a the sum of a Lorentzian function (XX emission line, red dashed line) and a Voigt profile (X_2 emission line, green dashed line) . The black dotted line identifies the center of the detection window.

of $g^{(2)}(0)$ up to a 50 % collection efficiency of the exciton PL intensity, with $g^{(2)}(0) = 0.29$ for a bandpass of 30 meV. Using such a bandpass would in turn enable us to perform measurements at lower excitation power density, reducing even more the contribution of the biexciton line. We thus expect our results to be amenable to improvement.

S7. POWER-DEPENDENT STARK SHIFT WITH POLAR GAN/ALN QUANTUM DOTS

As mentioned in the main text, III-nitride QDs are especially sensitive to variations in the laser pump fluence when compared to their non-polar counterparts. Each electron-hole pair captured by a dot gets separated by the built-in electric field and contributes, in turn, to its screening. Hence, the higher the excitation power density, the larger the number of trapped excitons per dot and the lower the Stark shift induced by this built-in field. The absolute magnitude of this shift is strongly dependent on the QD height. Indeed, electron-hole pairs trapped in large QDs experience a larger separation than in their smaller counterparts. The signature of this phenomenon is clearly observed in the PL spectra measured on QD ensembles at different excitation power densities shown in Fig. S9. The discrepancy between the $130\,\mathrm{mW\,cm^{-2}}$ (blue line) and $1.2\,\mathrm{W\,cm^{-2}}$ (black line) QD ensemble PL curves is larger for energies below $3.5\,\mathrm{eV}$, which is consistent with the increase in QD height. Similarly, it explains the observed narrowing in the PL peak FWHM with excitation power density (inset of Fig. S9) as the energy separation between low and high energy QDs becomes smaller. This power-dependent effect has an impact on ensemble TRPL measurements as for a given detection bandpass the luminescence from multi-excitonic recombination events (short-lived component of the decays) and the late luminescence from single exciton recombination (long-lived component of the decays) will obviously originate from different QD sets.

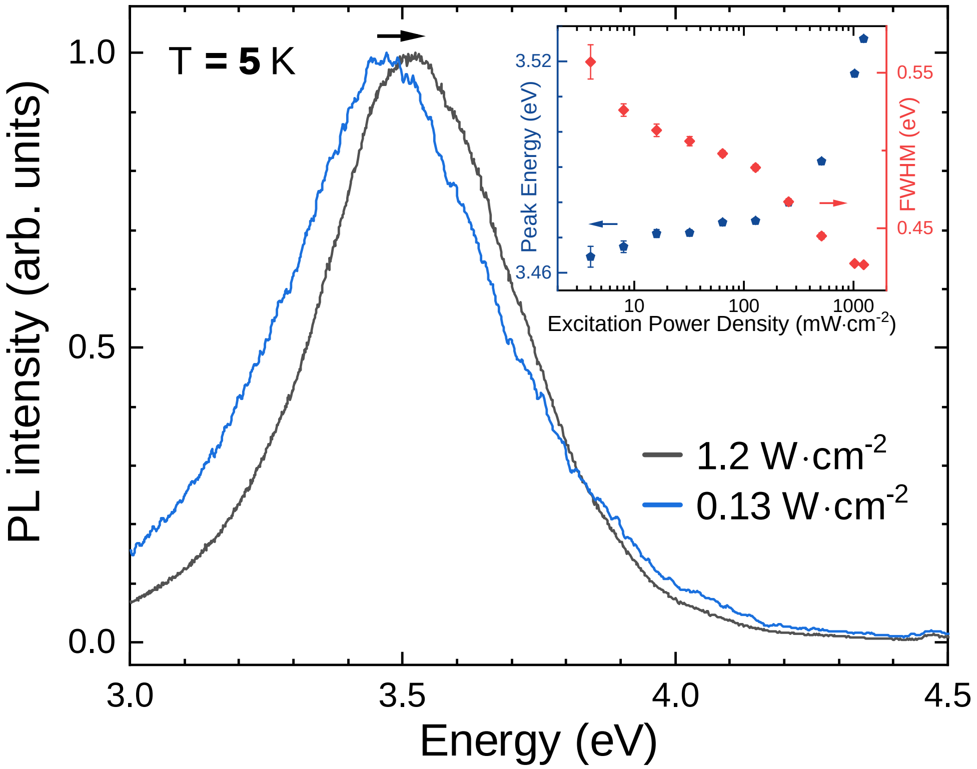

FIG. S9. Low temperature ($T = 5$ K) PL spectra of a polar GaN/AlN QD ensemble recorded at excitation power densities of 0.13 and 1.2 W cm^{-2}, respectively. The inset shows the blueshift of the peak energy and the FWHM narrowing of the PL spectra with increasing excitation power density.

S8. EVOLUTION OF THE BI-EXPONENTIAL PHOTOLUMINESCENCE DECAY WITH TEMPERATURE.

In the main text, we reported on the bi-exponential dynamic that governs the exciton recombination whatever the QD emission energy. Several scenarios were postulated to account for this behavior. One plausible explanation relies on a PL signal arising from the two distinct QD exciton energy bright states. In this framework, the fast decay time τ_S accounts simultaneously for the recombination of the high energy bright state B_2 and its relaxation toward lower energy states, i.e., toward the low energy bright state B_1 and the exciton dark states (cf. Fig. 2(a) of the main text). Since the long decay time τ_L remains constant with temperature (cf. Fig. 9 of the main text), it is hinting at the *a priori* negligible impact of phonon-mediated processes for the states exhibiting this recombination dynamic. In other words, τ_L is assumed to be mainly driven by the radiative recombination of B_1. To account

for this temperature-independence of τ_L, a quasi-equilibrium should take place between B_1 and the dark states that occurs on a much faster timescale than τ_L.

On the other hand, the weight of the fast component strongly depends on the refilling of B_2, which occurs through absorption of phonons with an energy matching the splitting between B_2 and lower energy states. Hence, the enhancement of the PL intensity originating from the high energy bright state can occur either through an increase in temperature or a reduction in the bright state energy splitting. The latter coincides with an increase in QD size and can be monitored through energy-dependent TRPL measurements.

In order to test these predictions, we extracted the long-lived PL component of the TRPL transients by integrating the associated long-lived mono-exponential fit over the whole PL intensity decay profiles, (as shown in Fig. 8 of the main text). In each case, this component was normalized to the PL intensity decay profile integrated over the whole raw data (for delays $\tau > 0$), hence leading to the intensity ratio denoted by the letter r in the main text. When reaching low excitation power densities, the contribution from multi-excitonic states to the TRPL transients is expected to vanish so that r should saturate ($r(\tau \to 0) \to r_0$). This is shown in Fig. S10 for QDs emitting at 3.80 eV. In the current framework, r_0 can be seen as a marker of the B_1 PL weight with respect to that of B_2, as shown by Eq. (6) in the main text. In other words, the weight of B_1 should increase together with r_0.

By monitoring a decrease in r_0 with decreasing QD emission energies (Fig. 10 of the main text), we showed that the long-lived decay contributes less to the PL of large QDs. This is in line with the expected decrease in the fine structure splitting with increasing QD size. From the ratios reported in Fig. S10, it appears now that r_0 is also decreasing with temperature for QDs emitting at 3.8 eV, as expected from the above-mentioned picture. The variation remains, however, relatively small (reduction of about 30 %) and additional results are required to confirm the global trend. Similar measurements led on high energy QDs would prove extremely valuable. Indeed, the large fine structure splitting characteristic of small QDs results in a strong discrepancy between B_1 and B_2 recombination rates. At 5 K, radiative recombination from the high energy bright state (X_2 line) is thus thermally suppressed and the X_1 prevails. On the other hand, X_2 dominates PL spectra as soon as B_2 refilling from lower energy states becomes thermally activated (cf. Fig. S5). From TRPL data, this should translate into a large ($r_0 \to 1$) and a small ($r_0 \to 0$) r_0 value at 5 K and 300 K, respectively. Given the very low luminescence signal issued from GaN/AlN QD

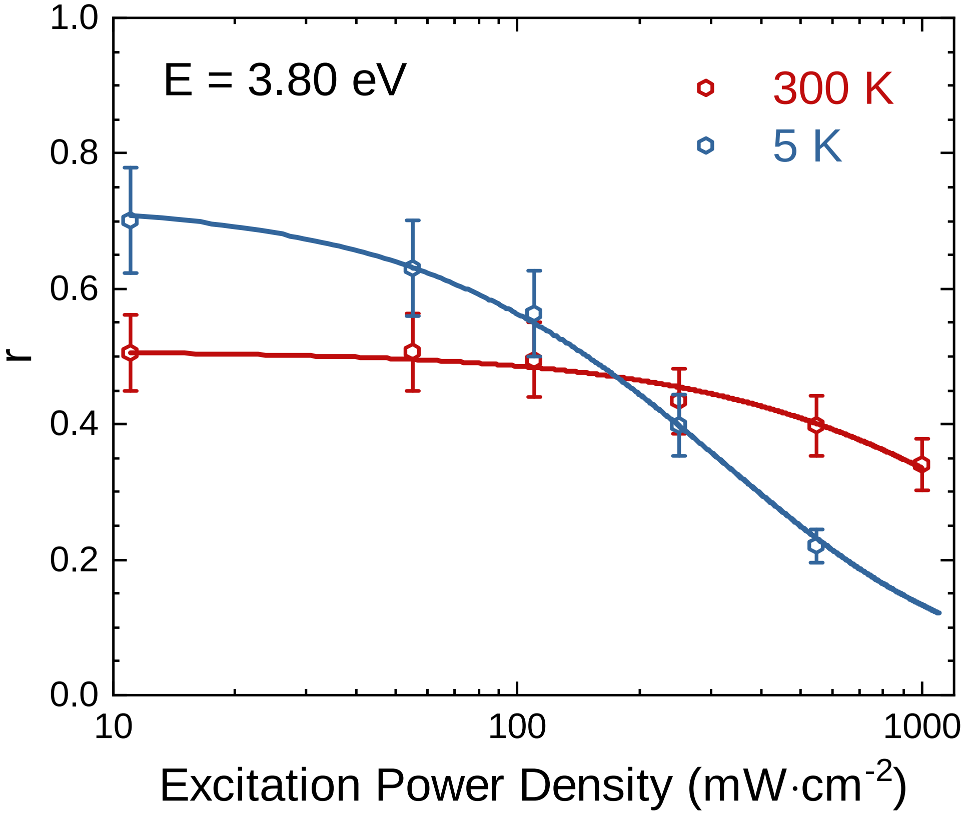

FIG. S10. Evolution of the intensity ratio r as a function of excitation power density for QDs emitting at 3.80 eV. Results collected at both 5 and 300 K are given. The solid lines serve as a guide to the eye. The uncertainties for the I_X component are estimated by varying the lower bound when integrating the mono-exponential fit. Thus, the lower bound was changed by about the duration of the fast decay, starting from $\tau = 0$, and the extreme values of I_X are used to set the error bars. Each data point corresponds to the mean integrated I_X value.

ensembles for energies above 4 eV, we could not record any exploitable TRPL transients at RT under very low excitation power densities. Nevertheless, we could extract a ratio $r = 0.25$ from the RT TRPL transient collected at 0.35 W cm^{-2} on QDs emitting around 4.25 eV (cf. Fig. S11). The latter ratio represents a substantial decrease from the $r = 0.82$ value obtained at 5 K under equivalent pumping conditions. Besides, r is observed to saturate faster at high temperature, as shown for QDs emitting near 3.80 eV (Fig. S10). Hence, the ratio r measured at 300 K for any excitation power density should be closer to the r_0 limit than its 5 K counterpart, i.e., $\frac{r}{r_0}(P_{\text{exc}}, 300\,\text{K}) > \frac{r}{r_0}(P_{\text{exc}}, 5\,\text{K})\ \forall\ P_{\text{exc}}$. At 5 K and for QDs emitting at 4.25 eV, we determined $\frac{r}{r_0}(0.35\,\text{W}\,\text{cm}^{-2}) > 0.9$. We can thus reasonably expect the ratio

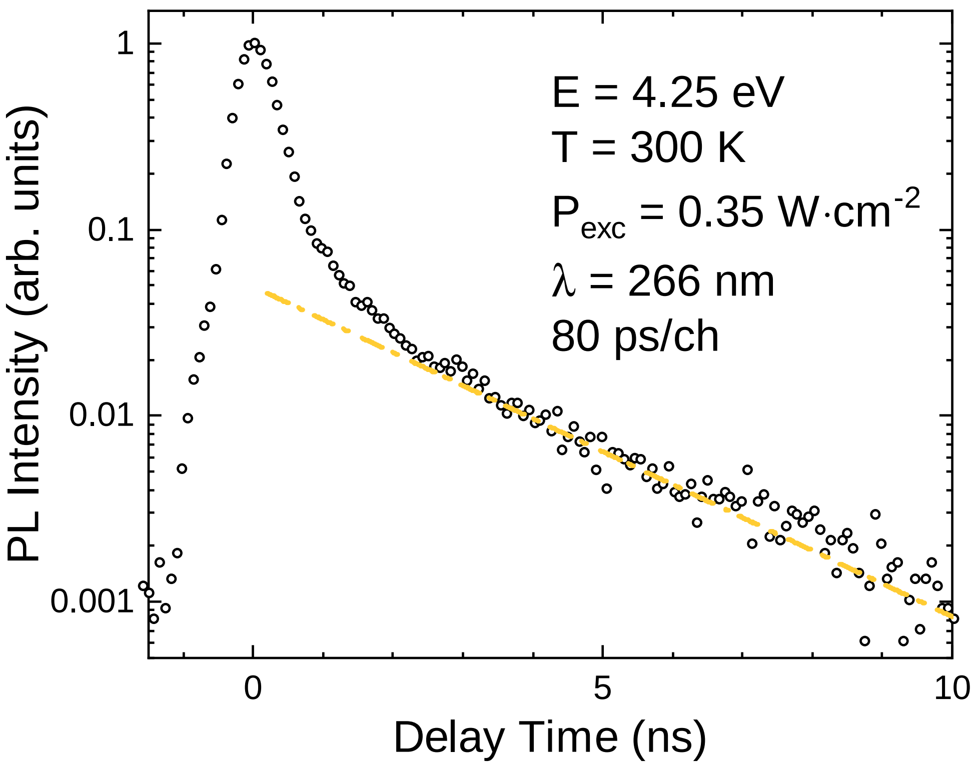

FIG. S11. Photoluminescence intensity decay of GaN QDs emitting at 4.25 eV recorded at 300 K for an excitation power density of $0.35\,\mathrm{W\,cm^{-2}}$. The tail is approximated by a single exponential with a decay time $\tau_{\mathrm{decay}} = 2.45\,\mathrm{ns}$ (yellow dash-dotted line).

$r = 0.25$ measured at RT under identical excitation power density ($P_{\mathrm{exc}} = 0.35\,\mathrm{W\,cm^{-2}}$) to differ by less than 10 % from r_0. As such, $r_0(300\,\mathrm{K}) < 0.3$ for QDs emitting at 4.25 eV, which scales way below the $r_0(5\,\mathrm{K}) = 0.9$ determined for similar QDs at cryogenic temperature. This confirms that the short-lived TRPL component is thermally activated.

S9. CATHODOLUMINESCENCE OF THE UNETCHED GAN/ALN QUANTUM DOT SAMPLE

A representative cathodoluminescence (CL) emission spectrum recorded at $T = 12\,\mathrm{K}$ using an acceleration voltage of 6 kV over an unetched area of the QD sample is shown in Fig. S12. The WL emits around 5.3 eV, which corresponds to an equivalent GaN thickness of ~ 1.5 monolayers. Let us note that the maximum CL intensity of the QD ensemble is shifted by about 200 meV with respect to the QD ensemble photoluminescence (cf. inset of

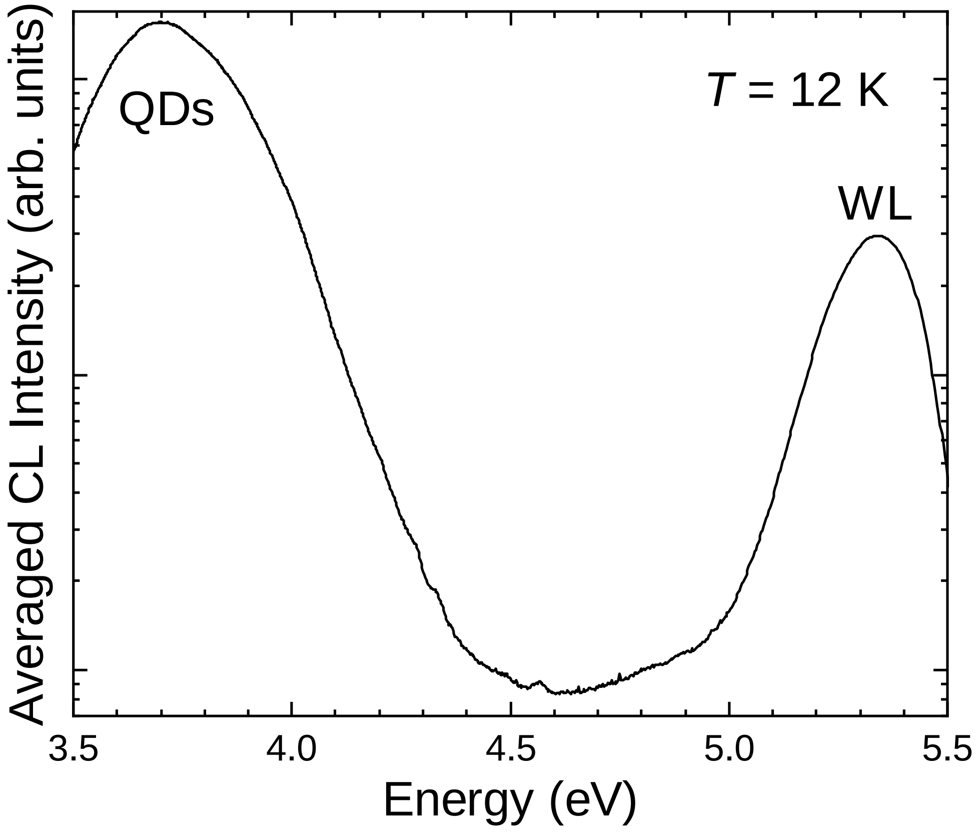

FIG. S12. Low temperature averaged CL spectrum measured using an acceleration voltage of 6 kV over an unprocessed area of the GaN/AlN QD sample. The peak at 5.3 eV corresponds to an ~ 1.5 monolayer-thick GaN WL.

Fig. 1**a** in the main text) as a result of the high carrier density generated in the present CL experiment, which leads to a screening of the built-in electric field. On the other hand, the WL emission remains marginally impacted even under high carrier injection because such very thin layer is almost insensitive to the quantum confined Stark effect.

[1] S. Tamariz, G. Callsen, J. Stachurski, K. Shojiki, R. Butté, and N. Grandjean, Toward Bright and Pure Single Photon Emitters at 300 K Based on GaN Quantum Dots on Silicon, ACS Photonics **7**, 1515 (2020).

[2] E. Rosencher and B. Vinter, in Optoelectronics (Cambridge University Press, Cambridge, 2002).

[3] D. Brunner, H. Angerer, E. Bustarret, F. Freudenberg, R. Höpler, R. Dimitrov, O. Ambacher, and M. Stutzmann, Optical constants of epitaxial AlGaN films and their temperature dependence, J. Appl. Phys. **82**, 5090 (1997).

[4] G. Hönig, G. Callsen, A. Schliwa, S. Kalinowski, C. Kindel, S. Kako, Y. Arakawa, D. Bimberg, and A. Hoffmann, Manifestation of unconventional biexciton states in quantum dots, Nature Communications **5**, 5721 (2014).

[5] M. Arita, F. Le Roux, M. Holmes, S. Kako, and Y. Arakawa, Ultraclean Single Photon Emission from a GaN Quantum Dot, Nano Letters **17**, 2902 (2017).

[6] R. Bardoux, T. Guillet, B. Gil, P. Lefebvre, T. Bretagnon, T. Taliercio, S. Rousset, and F. Semond, Polarized emission from GaN/AlN quantum dots: Single-dot spectroscopy and symmetry-based theory, Phys. Rev. B **77**, 235315 (2008).

[7] C. Kindel, Study on Optical Polarization in Hexagonal Gallium Nitride Quantum Dots, Ph.D. thesis, University of Tokyo (2010).

[8] T. Bretagnon, P. Lefebvre, P. Valvin, R. Bardoux, T. Guillet, T. Taliercio, B. Gil, N. Grandjean, F. Semond, B. Damilano, A. Dussaigne, and J. Massies, Radiative lifetime of a single electron-hole pair in GaN AlN quantum dots, Physical Review B **73**, 113304 (2006).

[9] A. Lohner, M. Woerner, T. Elsaesser, and W. Kaiser, Picosecond capture of photoexcited holes by shallow acceptors in p-type GaAs, Physical Review Letters **68**, 3920 (1992).

[10] A. Berthelot, I. Favero, G. Cassabois, C. Voisin, C. Delalande, P. Roussignol, R. Ferreira, and J. M. Gérard, Unconventional motional narrowing in the optical spectrum of a semiconductor quantum dot, Nature Physics **2**, 759 (2006).

[11] C. Kindel, G. Callsen, S. Kako, T. Kawano, H. Oishi, G. Hönig, A. Schliwa, A. Hoffmann, and Y. Arakawa, Spectral diffusion in nitride quantum dots: Emission energy dependent linewidths broadening via giant built-in dipole moments, Physica Status Solidi - Rapid Research Letters

8, 408 (2014).

[12] M. Hrytsaienko, M. Gallart, M. Ziegler, O. Crégut, S. Tamariz, R. Butté, N. Grandjean, B. Hönerlage, and P. Gilliot, Dark-level trapping, lateral confinement, and built-in electric field contributions to the carrier dynamics in c -plane GaN/AlN quantum dots emitting in the UV range, J. Appl. Phys. **129**, 054301 (2021).

[13] R. Brouri, A. Beveratos, J.-P. Poizat, and P. Grangier, Photon antibunching in the fluorescence of individual color centers in diamond, Optics Letters **25**, 1294 (2000).
